# Protein Thermodynamics Can Be Predicted Directly from Biological Growth Rates

**DOI:** 10.1371/journal.pone.0096100

**Published:** 2014-05-01

**Authors:** Ross Corkrey, Tom A. McMeekin, John P. Bowman, David A. Ratkowsky, June Olley, Tom Ross

**Affiliations:** Tasmanian Institute of Agriculture/School of Agricultural Science, University of Tasmania, Hobart, Tasmania, Australia; University of South Florida College of Medicine, United States of America

## Abstract

Life on Earth is capable of growing from temperatures well below freezing to above the boiling point of water, with some organisms preferring cooler and others hotter conditions. The growth rate of each organism ultimately depends on its intracellular chemical reactions. Here we show that a thermodynamic model based on a single, rate-limiting, enzyme-catalysed reaction accurately describes population growth rates in 230 diverse strains of unicellular and multicellular organisms. Collectively these represent all three domains of life, ranging from psychrophilic to hyperthermophilic, and including the highest temperature so far observed for growth (122°C). The results provide credible estimates of thermodynamic properties of proteins and obtain, purely from organism intrinsic growth rate data, relationships between parameters previously identified experimentally, thus bridging a gap between biochemistry and whole organism biology. We find that growth rates of both unicellular and multicellular life forms can be described by the same temperature dependence model. The model results provide strong support for a single highly-conserved reaction present in the last universal common ancestor (LUCA). This is remarkable in that it means that the growth rate dependence on temperature of unicellular and multicellular life forms that evolved over geological time spans can be explained by the same model.

## Introduction

Temperature governs the rate of chemical reactions including those enzymic processes controlling the development of life on Earth from individual cells to complex populations and spanning temperatures from well below freezing to above the boiling point of water [1]. The growth rates of unicellular and multicellular organisms depend on numerous processes and steps, but all are in principle limited by enzymic reactions [2]. This realization provides a link that bridges the gap between biochemistry and whole organism biology. By using the assumption of a single rate-limiting reaction step we show that we can describe the growth rate of diverse poikilothermic life forms. The temperature-dependent growth curves of poikilothermic organisms across their biokinetic ranges have a characteristic shape that may appear superficially to be U-shaped, but attentive examination shows them to be more complex. The history of previous approaches to describing these curves is extensive [3]–[6]. We use a model to describe the effect of temperature on biological systems that assumes a single, rate-limiting, enzyme-catalyzed reaction using an Arrhenius form that also allows for protein denaturation. The relative success of microbial strains within populations has been shown to be critically dependent on protein denaturation [7]. Earlier we presented such a model and fitted it to 95 strains of microbes [8]. In this work in addition to data on microorganisms, we also include data on the intrinsic growth rates for insects and acari obtained from life table analysis and find that these multicellular strains are also well described by the model. In total, we model 230 datasets (called strains herein) that cover a temperature range of 124°C. Notable amongst the modeled strains is the inclusion of hyperthermophiles active at the highest temperatures so far known for biological growth (121°C [9], 122°C [10]). The lowest temperature modeled was −2°C, below which growth rates cannot be reliably compared due to ice formation and the zone of thermal arrest. In this paper we address biological implications and results arising from examination of much more extensive data than previously used [8] and by grouping strains by their thermal optima rather than by taxonomy.

In essence, we model the growth rates of strains by assuming each strain is rate-limited by a single common enzyme which becomes denatured both at sufficiently high and at sufficiently low temperatures. The model uses growth rate data directly rather than modeling protein function. The model structure and definitions of the parameters are described in detail in the Materials and Methods. Briefly, we model the intrinsic growth rates for each strain (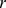) by using a function (equation 1) that describes a single, rate-limiting, enzyme-catalyzed reaction. The numerator of equation 1 has an Arrhenius form [11], [12], and the denominator describes the temperature-dependent denaturation of that enzyme. It requires eight parameters, four of which are assumed common to all life and are therefore held fixed (*viz*. the change in enthalpy and entropy for protein unfolding 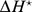, 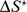, with associated convergence temperatures 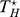, 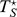, respectively), and four additional parameters for each strain that are associated with a rate-limiting enzyme (*viz*. scaling constant 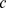; enthalpy of activation 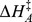; heat capacity change on denaturation 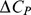; number of amino acid residues 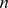). The model is fitted using a Bayesian hierarchical modeling approach that allows all data to be simultaneously considered and estimates obtained in a single run.

## Results and Discussion

We examined several alternative model structures that classified strains either: I) with all strains in a single group; II) into taxonomically defined groups that correspond to the three domains of life [13]: Bacteria, Archaea, or Eukarya; III) taxonomically, but allowing for multicellularity: Bacteria, Archaea, unicellular Eukarya, or multicellular Eukarya; IV) into thermal groups: psychrophiles, mesophiles, thermophiles, or hyperthermophiles; V) into thermal groups, except for fungi: psychrophiles, mesophiles, fungal mesophiles, thermophiles, or hyperthermophiles. Using a Bayes factor [14] approach we determined that the best performing model grouped the strains by thermal group, except for fungi, which were put into a separate group (model V). This model performed better than model IV, which combined the unicellular mesophilic fungal (Ascomycota) strains with the multicellular mesophilic taxa that included insects and acari.

Parameter estimates for the universal and thermal group parameters are given in Tables 1 and 2, respectively. Detailed parameter estimates for all strains are given in Table S1. The estimates obtained here extend those provided by earlier analyzes [8] in their breadth and especially in their improved precision due to the much larger data set. In particular, the two convergence temperatures (universal parameters) are now estimated to within 1.0 and 1.4 degrees, respectively.

**Table 1:** Posterior universal parameter estimates.

*Parameter*	*Mean*	*99% HPDI*
Enthalpy change (J/mol amino acid residue), 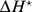	4874	(4846, 4913)
Entropy change (J/K), 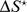	17.0	(16.9, 17.1)
Convergence temperature for enthalpy (K), 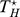	375.5	(375.1, 376.1)
Convergence temperature for entropy (K), 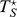	390.9	(390.3, 391.7)

Shown are the posterior means with 99% HPDI in parentheses.

**Table 2:** Posterior estimates of thermal group parameters.

*Thermal group*	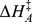 a		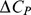 b		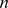 c	
Psychrophiles	48.6	(29.3, 59.8)	49.7	(46.5, 52.5)	388	(267, 531)
Mesophiles	75.3	(72.6, 79.1)	59.9	(59.6, 60.2)	422	(388, 457)
Ascomycota	39.7	(37.2, 42.0)	61.7	(61.5, 62.0)	340	(323, 356)
Thermophiles	71.3	(65.9, 77.6)	71.4	(70.0, 72.7)	180	(156, 205)
Hyperthermophiles	96.0	(79.7, 123.8)	96.9	(92.1, 102.8)	101	(66, 144)

a Enthalpy of activation (J/mol).

b Heat capacity change (J/K mol-amino acid-residue).

c Number of amino acid residues.

Shown are the posterior means with 99% HPDI in parentheses.

### Model fit

**Figure 1:**
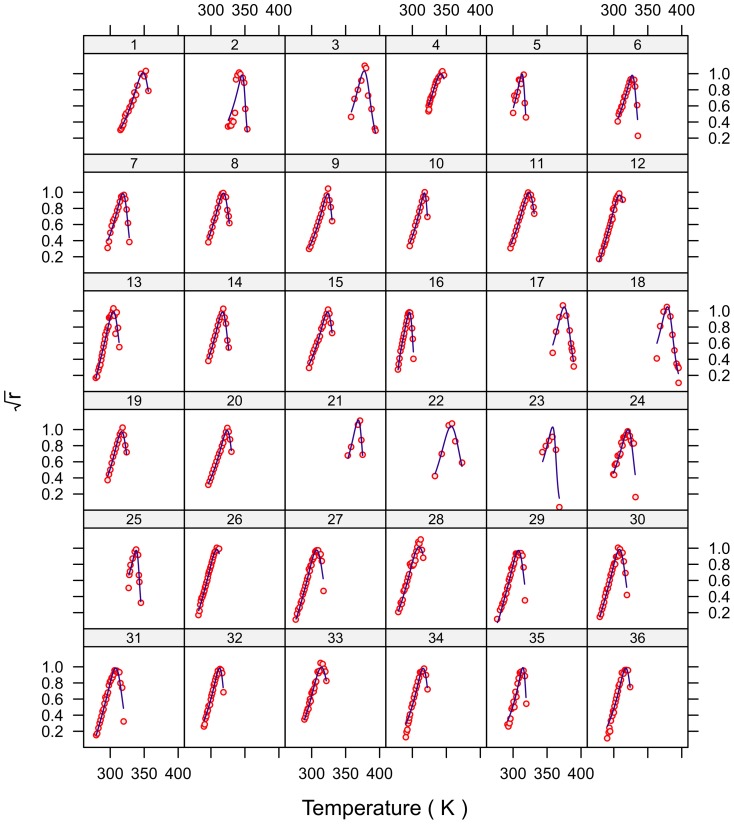
Fitted curves for strains 1–36.

**Figure 2:**
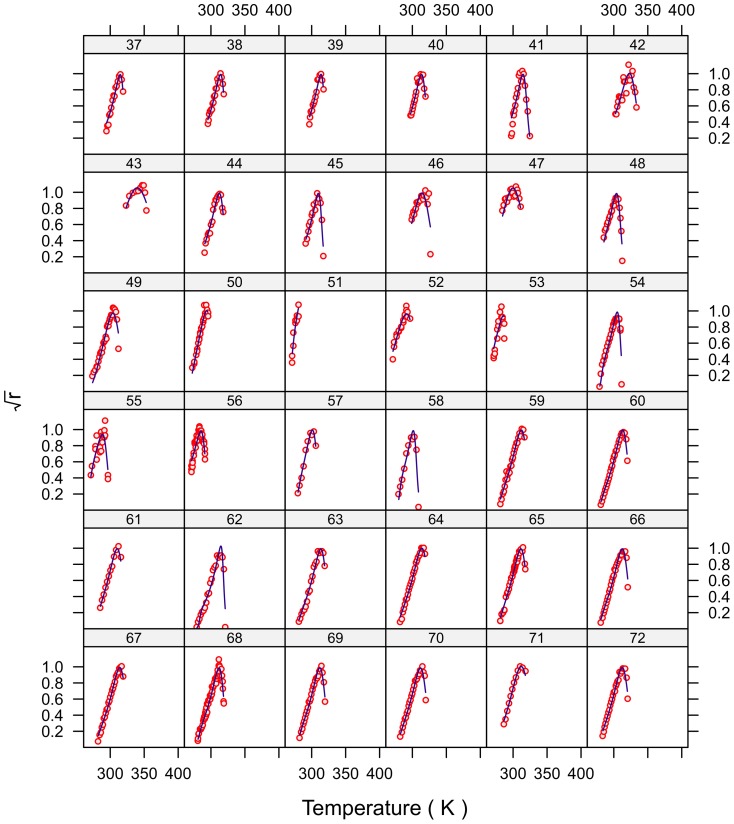
Fitted curves for strains 37–72.

**Figure 3:**
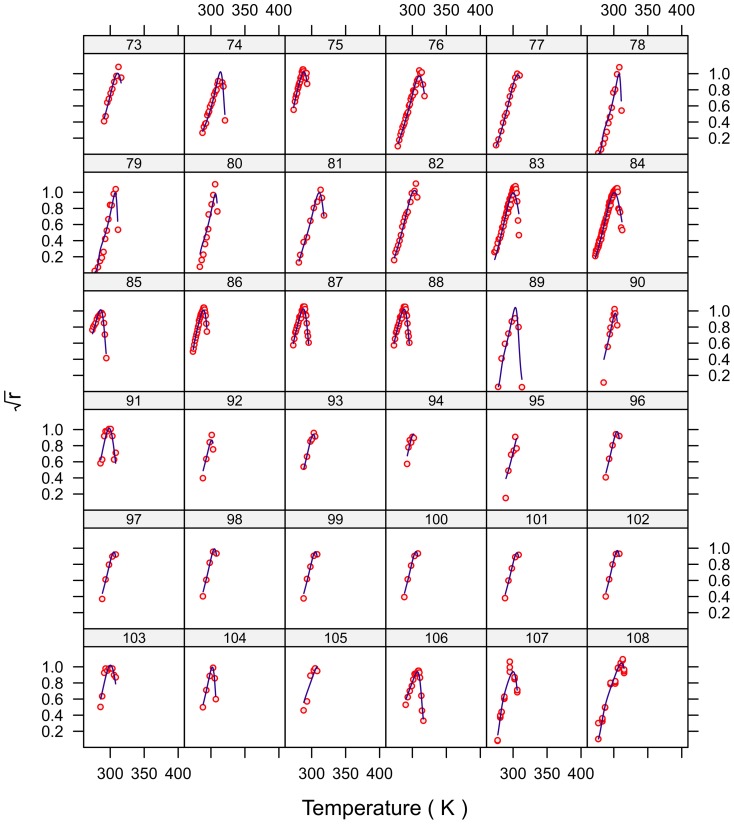
Fitted curves for strains 73–108.

**Figure 4:**
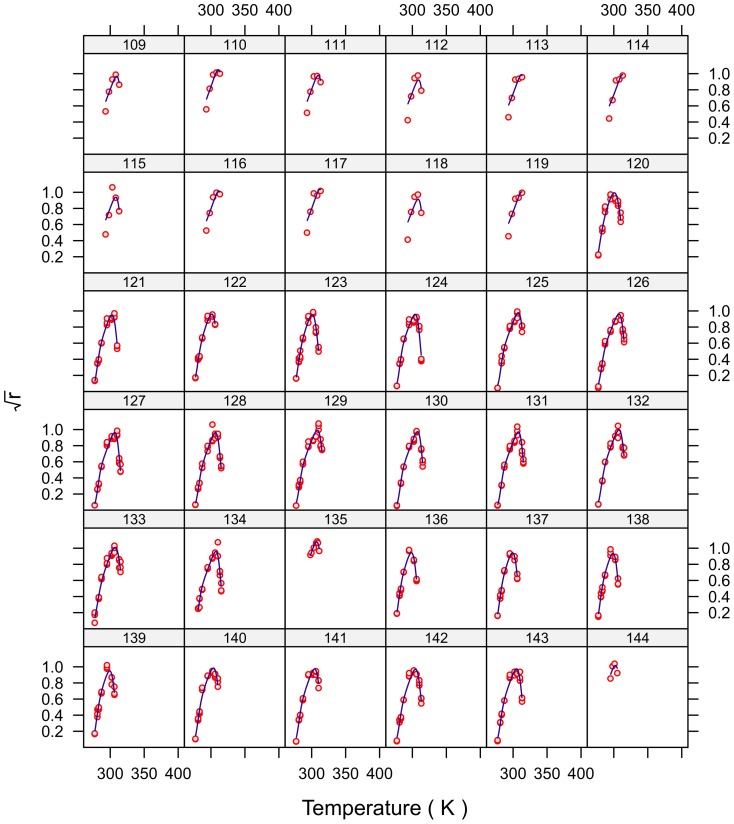
Fitted curves for strains 109–144.

**Figure 5:**
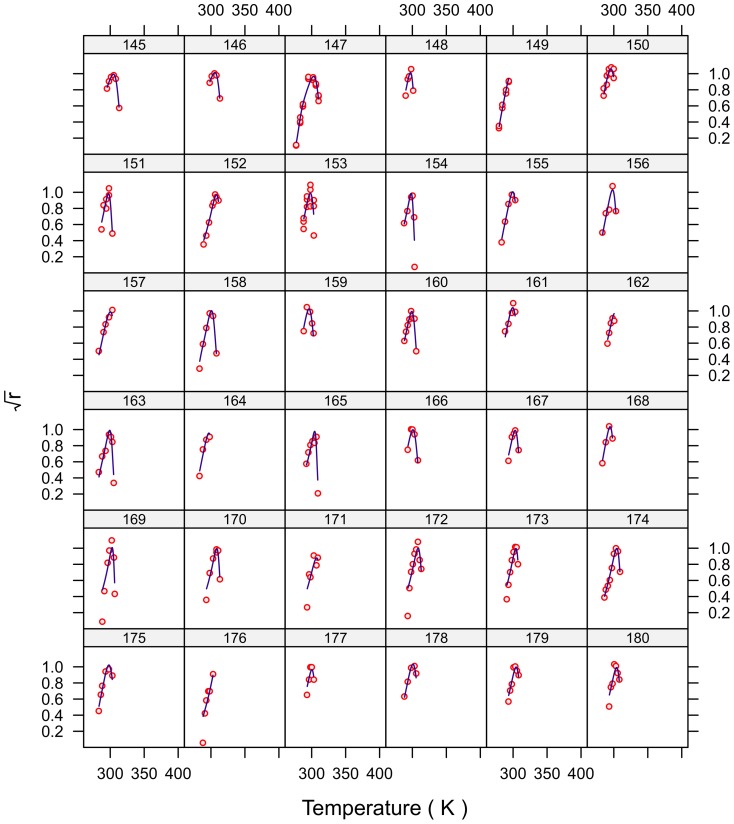
Fitted curves for strains 145–180.

**Figure 6:**
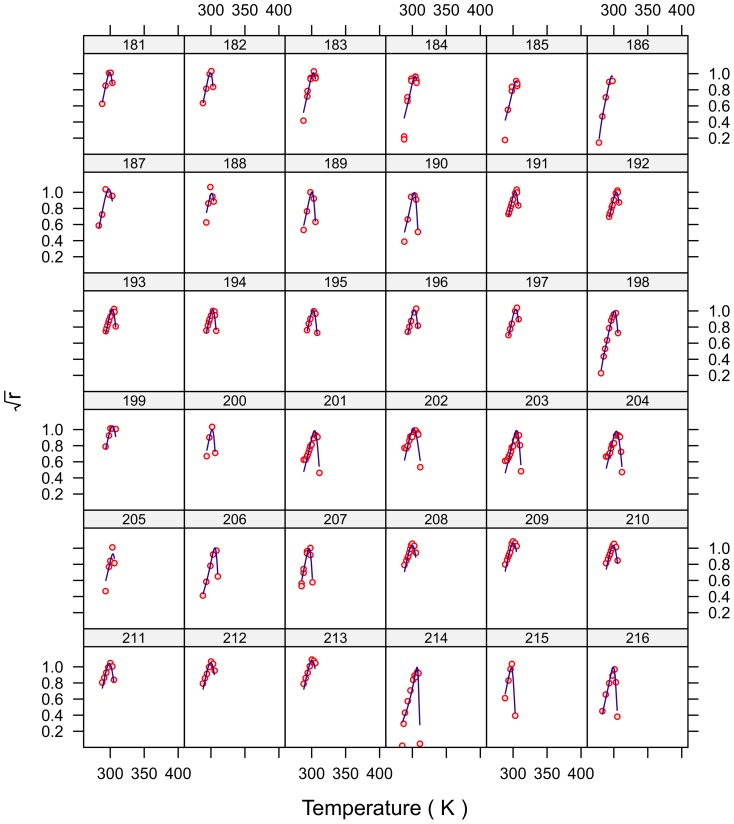
Fitted curves for strains 181–216.

**Figure 7:**
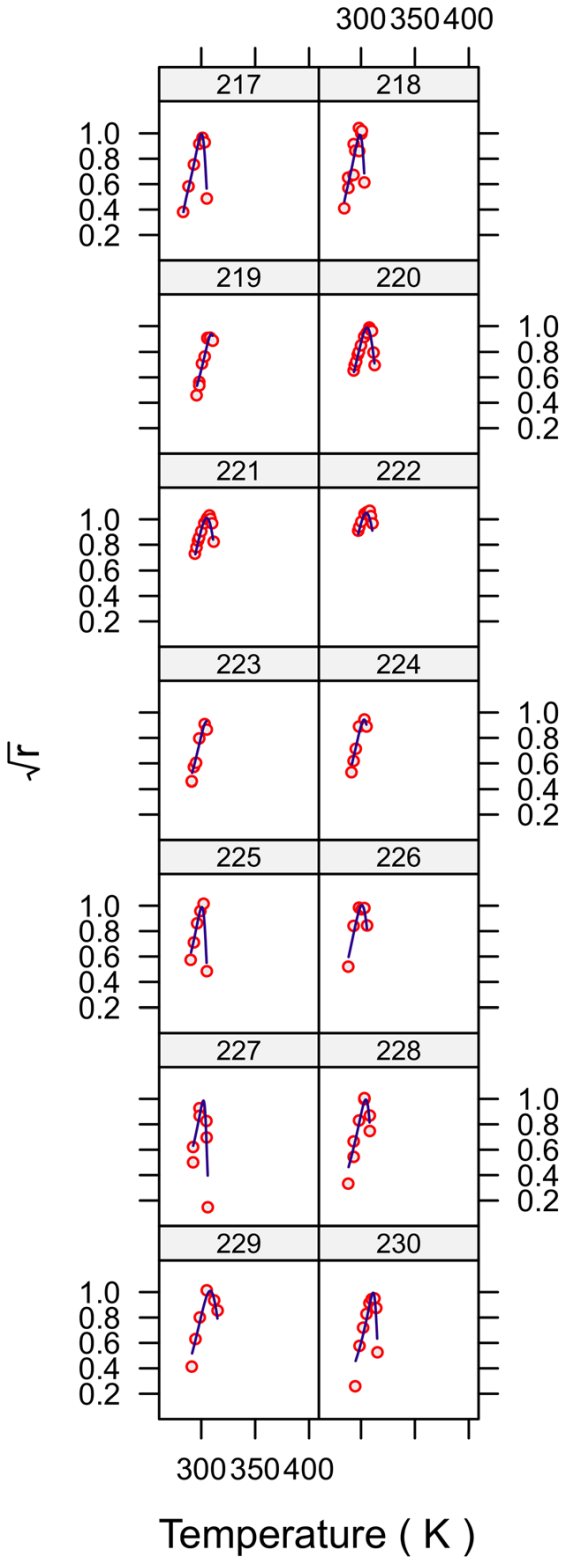
Fitted curves for strains 217–230.

The fits for all 230 strains are shown in Figure 1–7 and are excellent for almost all strains even including those with few data, and across the large temperature range spanned by the data sets. For example, strains 12 and 13 grew at temperatures as low as 280K while strains 17 and 18 grew at temperatures in excess of 390K.

### Thermodynamic relationships

**Figure 8:**
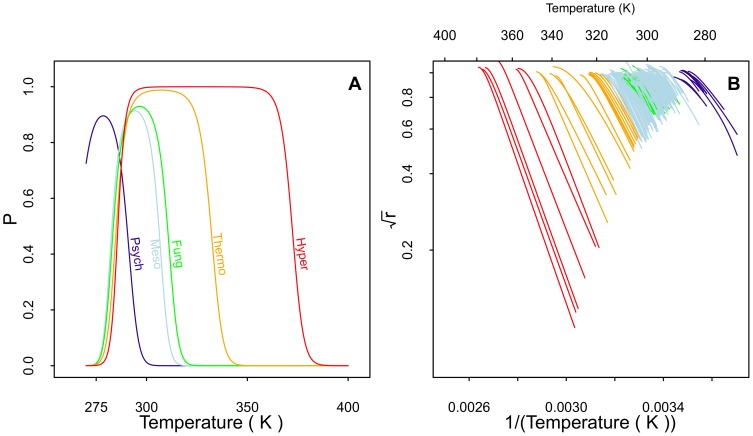
Probability of native state curves and portions of strain fitted curves between 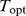 and 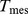. **A**: probability of native state curves for the thermal groups showing the flat-topped curves for the hyperthermophiles, reduced peak maximas for psychrophiles and Ascomycota, common lower temperature limit for the mesophiles, thermophiles, and hyperthermophiles, and displacement of the psychrophile curve to lower temperatures than the other domains. **B**: the portions of the fitted growth curves for all strains between 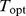 and 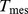 showing a trend for a broader gap in the more thermophilic strains. The plot uses a logarithmic scale on the vertical axis and the reciprocal of the temperature on the bottom horizontal axis.

The probability of the native (catalytically active) state for the thermal groups is shown in Figure 8A; we refer to the latter as native state curves [15] since they represent the proportion of the rate-controlling enzyme that is in the native conformation. The curves for the probability of the native state have lower peaks for psychrophiles, mesophiles, and Ascomycota, and the curves are taller and progressively flattened for thermophiles and hyperthermophiles. The higher and flatter peaks for the thermophiles and hyperthermophiles suggests protein stability over an increasingly extended temperature range. The lower peak levels for the lower temperature groups might be interpreted as reduced stability for psychrophile [16] and Ascomycota proteins [17]. The psychrophile native state curve is also shifted to the left of the other groups, which are all approximately aligned at the same lower temperature (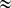
275K). The deviation of the psychrophiles below the other groups suggests that a mechanistic difference has evolved separating psychrophiles from the other groups.

The native state peak of each curve occurs at 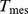 which is functionally dependent on 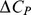 (Table 3). Also in Table 3, 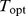, the temperature of maximal growth rate, tracks very closely the upper end of the native state curve so that the temperature difference between 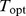 and the upper temperature of 50% stability (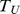) is very small for all groups, ranging from 2.5° for mesophiles to 4.2° for fungal mesophiles. In contrast is the difference between 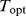 and the lower limit of the native state (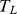) which increases from a modest 23°C for psychrophiles but reaches as high as 83°C for hyperthermophiles. Last, the difference of 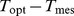 is virtually a constant for psychrophiles, mesophiles, fungal mesophiles (10°–11°), but dramatically increases for thermophiles (23°) and hyperthermophiles (44°; Figure 8B). These observations suggest that as the enzymes adapted to withstand higher and higher temperatures, their optimal thermal activity did not lag far behind, and they lost little of their ability to function at lower temperatures.

**Table 3:** Means of derived parameters.

*Thermal group*	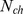 a	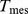 b	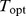 c	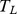 d	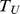 e	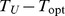	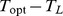	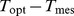
Psychrophiles	4.64	277	288	265	291	2.7	23	11
Mesophiles	5.08	294	305	283	307	2.5	23	11
Ascomycota	5.3	296	306	283	310	4.2	23	10
Thermophiles	6.35	307	330	285	332	2.5	45	23
Hyperthermophiles	8.64	325	369	286	372	2.4	83	44

a Average number of non-polar hydrogen atoms per amino acid residue.

b Temperature at which denaturation is minimized (K).

c Temperature at which growth is maximized (K).

d The lower temperature at which the putative rate-controlling enzyme is 50% denatured (K).

e The upper temperature at which the putative rate-controlling enzyme is 50% denatured (K).

**Figure 9:**
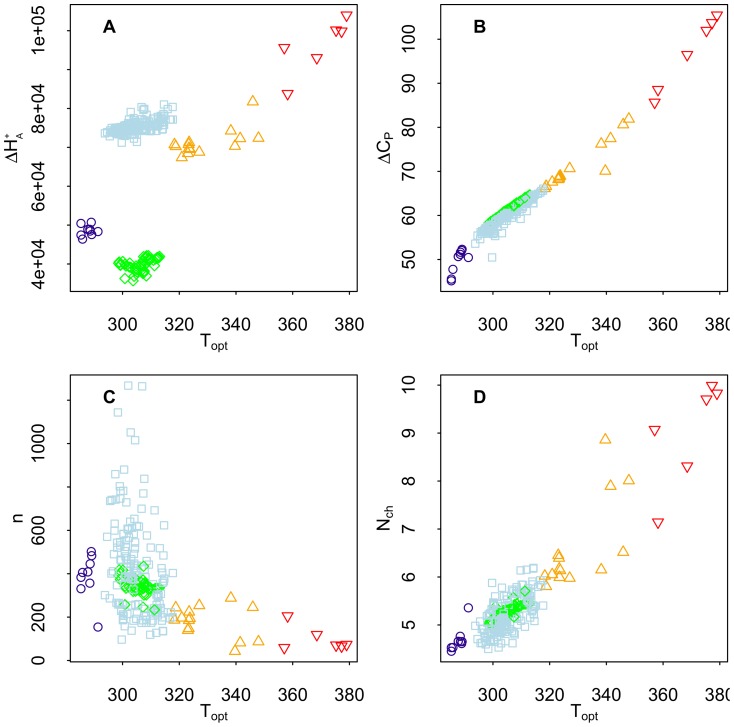
Relationships between thermodynamic parameters and 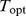. **A**: enthalpy of activation (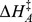) versus 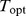. **B**: heat capacity change (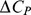) versus 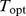. **C**: number of amino acid residues (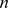) versus 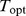. **D**: average number of non-polar hydrogen atoms per amino acid residue (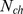) versus 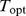.

We show in Figure 9A that the enthalpy of activation (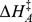) and in Figure 9B the heat capacity change (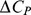) both generally increase with optimal temperature (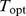). We can consider 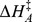 as relating to enzyme activity and 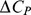 as relating to enzyme stability [18] as well as hydrophobicity of the putative rate-controlling enzyme [19]. The 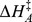 is smallest for Ascomycota followed by an increasing trend: psychrophiles, mesophiles/thermophiles, and then hyperthermophiles. The Metabolic Theory of Ecology [20], which describes metabolism and other biological processes in terms of an Arrhenius temperature dependence, explicitly assumes a constant enthalpy of activation (where it is called ‘activation energy’), although other work implies that it may not be invariant [21]. Our results indicate that for the majority of strains in our data, which are mesophiles and thermophiles, the enthalpy of activation is roughly constant with only a minimal increasing trend in these groups with increasing 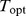, but for a broader range of strains the spread in the enthalpy of activation is much larger.

In the case of the Ascomycota, all strains considered were mesophilic and were consistent with some [17], [22]–[24], but not all [25], experimental data. As a check we calculated a separate analysis of data for another Ascomycota species. We fitted the thermodynamic model (equation 1) to growth rate data not used in the Bayesian model for the Ascomycota species *Aspergillus candidus* [26] using PROC NLIN from the SAS System, version 9.2. This was the same method used previously [15] and required several parameters to be held constant to achieve convergence. We fixed 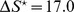, 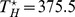, 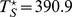 (these being the best estimates that we now have from the Bayesian runs). We obtained the following estimates for the remaining five free parameters: numerator constant 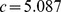, enthalpy of activation 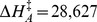, unfolding heat capacity change 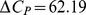, enthalpy change at the convergence temperature 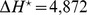 and number of amino acid residues 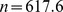. We note that the enthalpy of activation is very low, even lower than the values we have been getting for yeasts. The enthalpy change at the convergence temperature (4,872) is very close to the mean value estimated from the Bayesian run for that parameter, viz. 4,874. The 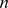 value of 617.6 is higher than the mean value obtained for that parameter from the Bayesian run for psychrophiles (388) and for yeasts (340), but we expect the value to be higher at the low temperature adaptation end of the temperature scale than at the thermophilic end of the adaptation scale, and that is the case. The heat capacity change for folding/unfolding of 62.2 is very close to that obtained for yeasts in this study.

The fungal proteins associated with the particular strains used in the Bayesian model may have low enthalpies of activation and, due to an inherent instability of yeast prion-type proteins, like psychrophilic proteins, are assisted by chaperones and chaperonins. Interestingly, their instability led to some workers suggesting that they are potentiators and facilitators of evolution [27]. In the case of the psychrophiles and hyperthermophiles, the apparent deviation of enthalpy of activation (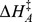; Figure 9A) below and above the mesophiles and thermophiles suggests the possibility that the rate-limiting reaction has been subject to adaptation for their respective environments.

In Figure 9C we predict that the number of amino acid residues (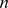) declines with the optimal temperature for growth (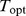). A negative correlation of protein length and optimal growth temperature has been reported [28], [29]. In Figure 9D the average number of non-polar residues per amino acid residue (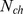) is predicted by the model to increase with optimal temperature (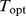), as has been observed experimentally for psychrophilic Archaea [16]. This is consistent with the observation that the more thermophilic proteins of Archaea have a greater hydrophobicity compared to mesophilic homologues [30], [31].

**Figure 10:**
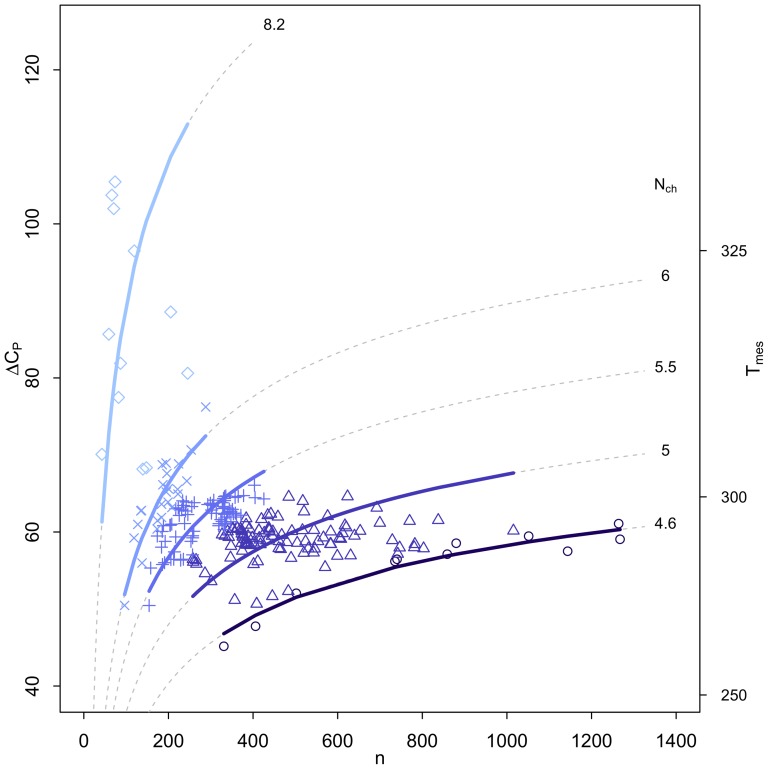
Relationship between thermodynamic parameter values 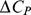, 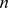 and 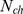. Shown is 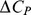 versus 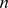 for all strains after partitioning the data into intervals based on 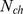. Each resulting set is indicated by different symbols and color shading, and for each Graziano *et al*'s predicted relationship [32] is plotted with the mean 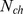 as labeled. Also shown is the 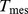 (on the right-hand axis) corresponding to the 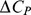 on the left-hand axis.

**Figure 11:**
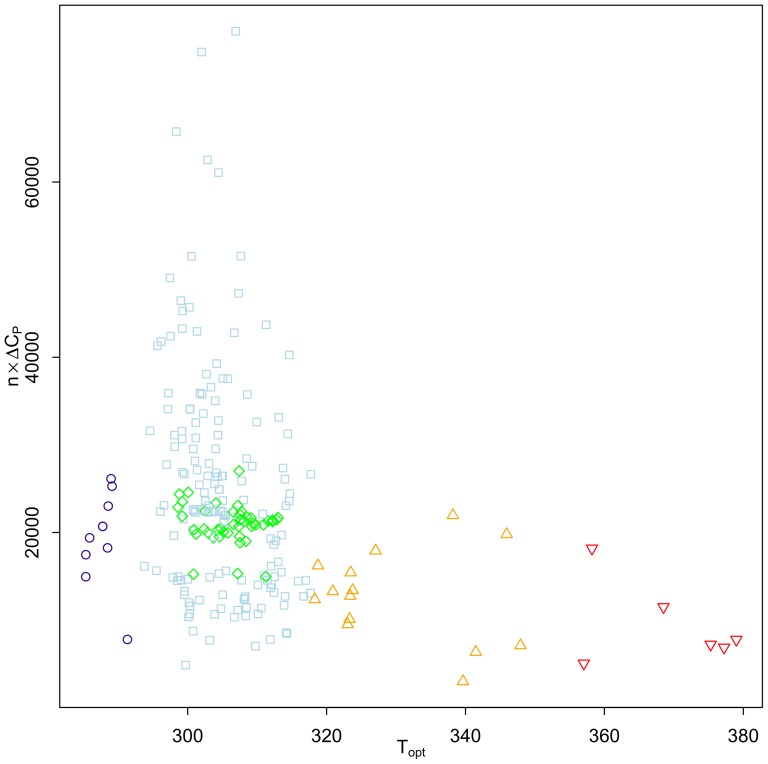
Total heat capacity change versus 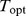. Shown is the total heat capacity change (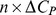) versus 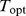. Colors and symbols are: psychrophiles: dark blue circles; Ascomycota: green diamonds; mesophiles: light blue squares; thermophiles: orange triangles; hyperthermophiles: red inverted triangles.

As noted above, we observed a trend in increasing 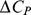 from psychrophiles to mesophiles (including Ascomycota) to thermophiles to hyperthermophiles. Also, there appears to be a negative correlation between 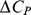 (per amino acid residue) and 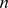 (Figure 9B, 9C)), illustrating that the relationship of these parameters can be complicated when examined with organism-level data. In Figure 10 we show that 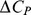 appears to decline as 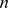 increases, but after partitioning the data into successive intervals of 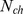 we see that within each interval they have a positive relationship. In Figure 10 we also show Graziano *et al*'s predicted relationship [32] as a visual guide, 
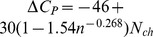
. The interpretation is that thermophilic proteins are more hydrophobic (larger 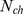) and that as 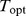 increases, the 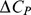, which is determined by the reorganization of water molecules around the polar and non-polar groups of the protein following denaturation, increases more rapidly as 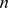, an index of the size of the protein, increases. This relationship is determined by the ratio of the buried and exposed surface of the proteins to avoid a close-packed core inaccessible to water molecules [32]. The total heat capacity change for the protein, given by 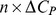, is shown in Figure 11 to decrease with 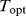. This is consistent with previously suggested mechanisms for stabilizing thermophilic proteins [33], [34].

### Stability-activity tradeoffs

Low temperature environments are constrained by low thermal energies and accordingly psychrophilic proteins have low enthalpies of activation, allowing biologically useful rates to be obtained at low temperatures. In the case of hyperthermophiles the environment is more demanding and therefore more stable proteins are predicted. These unfold more slowly [33] perhaps as a result of greater hydrophobicity [35] and an increased number of salt bridges [36], and also tend to be more highly expressed [37]. Many proteins also rely on assistance from molecular chaperones including the heat shock family of proteins, or the more complex structures known as chaperonins, to encourage correct protein folding and to rescue and repair misfolded proteins [38]. It is thought that proteins are maintained by evolution to be only as stable as needed for their environment [39], [40], though their active centers are optimized to be maximally active at different temperatures [41].

Thermophilic proteins tend to be more stable against unfolding than their mesophilic equivalents [37]. Stability is achieved by an increase in enthalpic forces at higher temperatures while at lower temperatures proteins are more flexible becoming dependent on entropic forces [16], [36], [37]. At very low temperatures psychrophilic proteins are more flexible and less stable [18], also depending on chaperones, but to control cold denaturation [42]. It has been suggested that the balance of stability and activity arising from entropic and enthalpic forces is important for protein function [43], while in evolution, it is stability that is conserved [44]. Hyperthermophilic proteins are more slowly evolving than their mesophilic equivalents [31], [37] presumably because mutations in thermophilic proteins would have more deleterious impacts [45] and would not be perpetuated.

Hyperthermophilic proteins can be less kinetically sensitive to temperature [46], an effect congruent to that described here. A notable example is serum albumin, which is promiscuously catalytic, stable up to 150°C, and is largely homologous within vertebrates [47]. In other words, the more robust enzymes in thermophiles and hyperthermophiles are stabilized over a broader temperature range than in mesophiles and psychrophiles. While we obtain this effect from modeling organism intrinsic growth data, it is found in protein denaturation curves of individual proteins. For example, denaturation curves of phosphoglycerate kinases from the thermophilic bacterium, *Thermus thermophilus*, have been found to be almost flat over a 60°C range whereas those from yeasts were strongly temperature-dependent [48]. The trimeric protein CutA1 from the hyperthermophile *Pyrococcus horikoshii* [49] is more stable at all temperatures above 0°C than its thermophilic and mesophilic equivalents. The CutA1 protein is universally distributed in bacteria, plants and animals [50]. We suggest that there may be many other hyperthermophilic proteins still to be found in organisms with lower temperature optima.

### Unicellularity and multicellularity

The model fits unicellular specific growth rates [51] and intrinsic growth rates in the case of multicellular organisms derived by life table analysis [52]. The two rates are comparable since both describe the maximum growth rate after allowing for the mortality rate. We refer to them both as growth rates. A distinction between them is that the growth rate of multicellular organisms results from a more complicated sequence of events. However, the proportion of the time spent in particular developmental stages, such as pupa in insects and nymphs in mites, does not change with temperature since they depend equally on the temperature dependence of cell division [53]. In addition, within multicellular metazoan organisms there are control cells (thermosensory neurones) that are specialized in sensing heat shock and act to trigger an orchestrated hierarchical response to temperature change throughout the organism [54]. The remarkable implication of the excellent model fits is that the rate of biological growth at a given temperature, considered as a proportion of the maximum possible rate for a strain, whether in unicellular or multicellular organisms, is ultimately limited by the thermodynamics of enzyme reactions.

### The nature of the rate-limiting reaction

While the model performs excellently, both in terms of its general consistency with protein biochemistry and in the good fits obtained, some predictions do not fully agree with thermodynamic expectations and there exists the possibility that the underlying mechanism may be more complex than a single, rate-limiting, enzyme-catalyzed reaction. Nevertheless, the model underlines the importance of thermodynamics in biological processes especially those relating to the interaction between proteins and water molecules, which in turn may depend on the properties of water itself [55]. But if it does take the form of a single reaction then we can speculate on its nature. A mechanism by which cells control denaturation may be suggested by consideration of protein chaperones. Some examples are DnaK (Hsp70) and DnaJ (Hsp40) and the bacterial chaperonins GroEL and GroES [56]. Such systems act during *de novo* folding and to refold unfolded substrate proteins [38]. They are triggered by the inflated exposure of hydrophobic groups in the unfolded proteins [38]. GroEL and GroES function together to create an Anfinsen hydrophilic cage containing charged residues that accumulate ordered water molecules, causing the substrate protein to bury its hydrophobic residues and refold into its native state [56], [57]. The rate at which the GroEL and GroES function proceeds is controlled by ATP hydrolysis [58]. If heat shock proteins represent the rate-limiting step, the rate at which they function must be the critical factor. Those chaperones that are responsible for *de novo* folding and refolding are ATP-dependent [56]. Expression of important chaperones (GroEL, GroEL, GrpE, DnaK) seem to become silent as bacterial cells die from sudden thermal stress [59]. Therefore, we hypothesize that the rate-limiting may be linked to a process leading to or directly linked to protein folding. The modeled value of 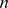 varies 4-fold (Table 2) suggesting the reaction could take different forms in different strains linked to their temperature preference. Reactions potentially include a range of important enzymes either enacting or supporting protein folding with denaturation of the reaction leading to inhibition of the broad protein folding process. Possible examples include trigger factor [60], peptidylprolyl isomerases (the slow step in protein folding) [61], protein disaggregation [62] and maintenance of ATP availability to the folding system [63], [64].

Notably, we find that the predicted temperature of maximum protein activity increases with optimal temperature but at a lesser rate (Table 3). The pattern implies that the range of thermal activity for the rate-controlling step in hyperthermophiles has a much larger potential range than in thermophiles, and these in turn larger than in mesophiles. We propose that the remarkable occurrence of thermophilic proteins such as serum albumin and CutA1 in non-thermophilic organisms may be examples of such a phenomenon. The model provides strong support for a single reaction system common to all life and, therefore, must have been strongly conserved since the time of the last universal common ancestor (LUCA). The question of a hyperthermophilic LUCA remains unresolved [65]–[70] and while we do not speculate on the LUCA's nature, the suggestion of a metabolic commonality in the form of a highly conserved rate-limiting reaction may prompt further considerations on this issue.

## Conclusions

1. Our focus has shifted away from domains, and towards thermal adaptation groups to which all life belongs, as it is adaptation to temperature, and not taxonomy, that is the factor of importance in explaining the variation among data sets.
2. Significantly, these results are obtained without any use of protein data, but only by growth rate data from unicellular and multicellular organisms, thereby bridging the gap between biochemistry and whole organism biology.
3. Using growth rate data that describe how quickly unicellular or multicellular populations grow under non-limiting conditions, we obtain estimates of thermodynamic parameters for protein denaturation consistent with the published literature on the physiology of organisms.
4. With this approach, we can now obtain relationships between these thermodynamic parameters that were previously identified from protein chemistry experiments.
5. As we now have a universal model that fits population growth data for organisms that can be prokaryotic or eukaryotic, as well as unicellular or multicellular, the organisms thermal adaptation position (i.e. whether it is a psychrophile, mesophile, thermophile or hyperthermophile) and, if a mesophile, whether it is single-celled or multi-celled, is sufficient to predict reliably its relative rate response to temperature.
6. We also advance the modeling approach by updating the universal parameters using adaptive direction sampling instead of Metropolis-coupled MCMC that we previously used [8], resulting in a greatly reduced run-time that will make further model development much more feasible.
7. We find it remarkable that unicellular and multicellular life forms that evolved over at least 3 billion years can be described by the same temperature dependence model.

## Methods

### Data

The data summarized in Table S1 comprised 3,289 records of intrinsic growth rates (or rates of metabolism in some cases) of 230 strains from 31 Bacteria, 20 Archaea, and 77 Eukarya species. They covered a temperature range of 271.2–395.3K (−1.95–122.15°C). They included 10 psychrophiles (e.g. *Gelidibacter* sp.), 157 mesophiles (e.g. *Escherichia coli*), 43 mesophilic fungi (Ascomycota; e.g. *Monascus ruber*), 14 thermophiles (e.g. *Acidianus brierleyi*), and 6 hyperthermophiles (e.g. *Methanopyrus kandleri*). The thermal groups are defined below. Not all domains of life were represented in all thermal groups; Eukarya, in particular, is thought to have an upper limit of 60°C [71]. The organisms are very diverse and include acidophiles (e.g. *Ferroplasma acidiphilum*), halophiles (e.g. *Haloarcula vallismortis*), haloalkaliphiles (e.g. *Natronococcus occultus*), an alga (*Chlorella pyrenoidosa*), as well as multicellular organisms including insects (e.g. *Clavigralla tomentosicollis*), acari (e.g. *Amblyseius womersleyi*), and a collembola (*Paronychiurus kimi*).

### Model structure

Below, we refer to the observed growth rate as 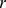 and the modeled growth rate as 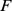. The model shown in equation 1 below assumes that the growth rate is governed by a single, enzyme-catalyzed reaction system that is limiting under all conditions. In the equation the quantity 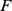 is the predicted rate given the temperature and the values of the parameters. The numerator (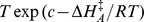) is essentially an Arrhenius model that describes the rate of the putative enzyme-catalyzed rate-controlling reaction (RCR) as a function of temperature while the denominator models the change in expected rate due to the effects of temperature on the conformation and, hence, catalytic activity of the putative enzyme catalyzing that reaction.
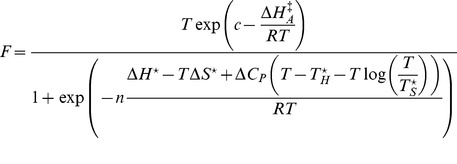
(1)


In equation 1: 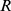 is the gas constant (8.314 J/K mol); 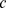 is a scaling constant; 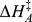 is the enthalpy of activation (J/mol); 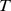 is the temperature in degrees Kelvin; 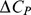 is the heat capacity change (J/K mol-amino acid-residue) upon denaturation of the RCR; 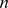 is the number of amino acid residues; 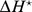 is the enthalpy change (J/mol amino acid residue) at 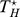, the convergence temperature for enthalpy (K) of protein unfolding; 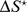 is the entropy change (J/K) at 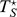, the convergence temperature for entropy (K) of protein unfolding.

We derive several further quantities. One is the average number of non-polar hydrogen atoms per amino acid residue [32]: 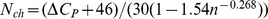. Another is 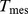, the temperature at which denaturation is minimized [15]. This temperature provides an index of temperature adaptation of the organism and was calculated as 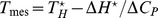. Last, there is the optimal temperature for growth, 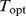, which was calculated numerically from the fitted growth rate curves.

We allowed four parameters to have values specific to each strain: 
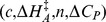
. We assumed the strain parameters to be Gaussian distributed with means specific to their grouping within the model. We constructed alternative groupings of the strain parameters, which we labeled: I, II, III, IV, and V. For model I we only used a single group to which all the strains belonged. In model II we allocated the strains to one of the taxonomic domains Bacteria, Archaea, or Eukarya. Model III was the same as model II except that we split Eukarya into unicellular and multicellular groups. Model IV grouped strains according to the four thermal groups given below, but ignored the taxonomic domains to which the strains belonged. Allocation to the thermal group followed an initial model fit from which we obtained estimates of 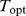. The strains were then allocated into the thermal groups as follows: psychrophile: 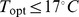; mesophile: 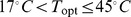; thermophile: 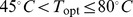; hyperthermophile: 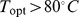. Model V was the same as model IV but included an additional group for the Ascomycota since exploratory work indicated they may differ from the other groups.

The remaining parameters (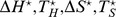) described protein thermal stability limits [72]–[74] and were not expected to depend on the individual biochemistry of each strain. Indeed, our earlier study [8] and exploratory work supported this conclusion. Accordingly, in the model structure, these values were assumed common to all strains. We refer to these as universal parameters.

To control the variance homogeneity we worked on the square root scale [75]–[77]. We assumed that the square root of the observed growth rate had a Gaussian distribution with a mean given by the square root of the modeled value, 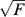, and with an unknown precision (reciprocal variance), 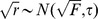.

The data were standardized for each strain by dividing by the maximum rate for each strain so that all the standardized rates were in the range 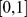. This ensured that the rates were not size-dependent. A subsequent standardization was conducted following an initial model fit by dividing the observed data for each strain by the fitted maximum rate for that strain. These model-scaled data were then used in subsequent analyzes. This procedure meant that the influence of the 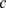 parameter was effectively removed from the model.

### Implementation

We used a Bayesian approach to allow for uncertainty in measurement and parameters to be incorporated in a natural way through the appropriate prior specification. We assigned normal priors to the strain parameters in which the means were specific to the taxonomic group for models I, II, and III, or thermal group for models IV and V: 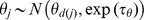, in which 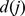 is the taxonomic or thermal group for strain 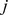. The 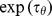 is the strain precision and models the variation between the strain parameters about the 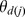 parameters. The taxonomic and thermal group means and the 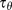 were assigned uniform priors with limits informed by the biochemistry literature with the exception of 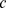 which was assigned a vague prior. The universal, thermal group and taxonomic group parameters were each assigned a uniform prior with limits informed by the biochemistry literature. Finally, the observational precision was assigned a gamma distribution, 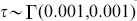. Prior specifications are documented in Table 4. Inference was obtained in the form of posterior means and variances using Markov Chain Monte Carlo (MCMC) simulation [78]. We chose to update the parameters of each strain as a block using Haario updates [79]. We also used Haario updates for each set of taxonomic or thermal group mean parameters and the strain parameter precisions. For the universal parameters we used adaptive direction sampling [80] combined with a low probability stepping-stone proposal [81]. This resulted in a much reduced run-time compared to previous work [8]. The models were run for 1,000,000 iterations and the last 50% of iterations retained for further analysis. We compared the models using Bayes factors [14] obtained using a pseudo-prior approach [82]. There was a clear separation between the five models with model V being preferred over the other four models with Bayes factors of: 1.0e9, 7.0e7, 9.1e2, and 9.9e4. We therefore continued only with model V. We summarized parameters using posterior means, standard deviations, and 99% highest posterior density intervals (HPDI). A 99% HPDI is the shortest interval that contains a parameter with 99% probability.

**Table 4:** Priors for model parameters.

*Parameter (with supporting literature references)*	*Priors*
Scaling constant	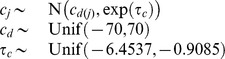
Enthalpy of activation [4], [83]–[92]	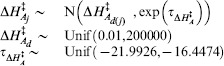
Heat capacity change [93], [94]	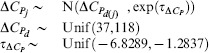
Number of amino acid residues [95], [96]	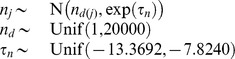
Enthalpy change at convergence temperature [97]	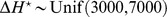
Entropy change at convergence temperature [97]	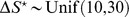
Convergence temperature for enthalpy [94], [97], [98]	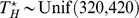
Convergence temperature for entropy [97]	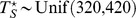

Shown are the prior distributions which are either Gaussian or uniform distributions. The parameters of the Gaussian distributions are their means and precisions (reciprocal variances). Strain level parameters are subscripted by 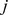, taxonomic or thermal group parameters by 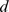, and membership of strain 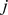 in group 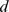 by 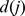.

## Supporting Information

Table S1 **Posterior strain parameter estimates showing means and standard deviations in square brackets.**
(PDF)
