# Supplementary material for: Protein Thermodynamics Can Be Predicted Directly from Biological Growth Rates
Source: PLoS One. 2014 May 1;9(5):e96100. doi: 10.1371/journal.pone.0096100 (PMC4006894; doi:10.1371/journal.pone.0096100)
Supplement: Table S1 — Posterior strain parameter estimates showing means and standard deviations in square brackets. (PDF) [file pone.0096100.s001.pdf]

Table S1: Posterior strain parameter estimates showing means and standard deviations in square brackets.

Code[a]	D.[b]	Taxa[c]	T.G.[d]	Strain name	Lit.[e]	Smp.[f]	$\Delta H_A^{\ddagger}$[g]	ΔC_P[h]	n[i]	N_{ch}[j]	T_{mes}[k]	T_{opt}[l]
1	A	Cr	T	*Acidianus brierleyi*	[1]	19	72.4 [2.48]	81.9 [0.94]	86.8 [11.9]	8.0 [0.24]	316.0 [0.71]	347.9 [0.63]
2	A	Cr	T	*Acidianus brierleyi*	[1]	14	81.7 [2.83]	80.6 [0.09]	245.5 [7.5]	6.5 [0.03]	315.0 [0.07]	345.9 [0.11]
3	A	Cr	H	*Strain 121*	[2]	10	99.9 [8.32]	103.7 [0.62]	66.3 [1.3]	10.0 [0.06]	328.5 [0.27]	377.2 [0.26]
4	A	Cr	T	*Sulfolobus metallicus*	[1]	20	72.3 [2.78]	77.5 [1.46]	82.0 [19.3]	7.9 [0.43]	312.6 [1.28]	341.5 [1.51]
5	A	Eu	M	*Ferroplasma acidiphilum*	[1]	11	76.3 [1.30]	64.0 [0.06]	517.5 [21.5]	5.2 [0.02]	299.4 [0.07]	313.1 [0.14]
6	A	Eu	T	*Ferroplasma cyprexacervatum*	[1]	20	68.8 [2.15]	70.7 [0.12]	253.6 [9.6]	6.0 [0.03]	306.5 [0.12]	327.1 [0.18]
7	A	Eu	T	*Haloarcula vallismortis*	[3]	17	70.2 [2.06]	66.6 [0.14]	243.4 [8.5]	5.8 [0.02]	302.3 [0.15]	318.8 [0.17]
8	A	Eu	T	*Halobaculum gomorrense*	[3]	17	70.7 [2.12]	66.1 [0.20]	186.8 [7.2]	6.0 [0.03]	301.8 [0.23]	318.3 [0.18]
9	A	Eu	T	*Halococcus morrhuae*	[3]	18	71.3 [1.97]	68.8 [0.15]	224.0 [11.1]	6.0 [0.04]	304.7 [0.15]	323.5 [0.19]
10	A	Eu	M	*Haloferax volcanii*	[3]	14	77.1 [1.26]	66.1 [0.10]	403.2 [25.6]	5.4 [0.04]	301.7 [0.11]	317.7 [0.20]
11	A	Eu	T	*Halogeometricum borinquense*	[3]	19	70.8 [2.11]	68.3 [0.31]	147.9 [8.9]	6.4 [0.05]	304.2 [0.32]	323.3 [0.23]
12	A	Eu	M	*Halorubrum lacusprofundi*	[4]	25	75.5 [1.24]	60.4 [0.26]	173.3 [9.7]	5.8 [0.04]	294.8 [0.34]	308.3 [0.21]
13	A	Eu	M	*Halorubrum lacusprofundi*	[4]	26	75.7 [1.24]	59.5 [0.13]	257.1 [6.6]	5.4 [0.01]	293.6 [0.18]	304.5 [0.09]
14	A	Eu	M	*Halorubrum saccharovorum*	[3]	16	77.2 [1.25]	65.5 [0.12]	221.6 [7.1]	5.8 [0.02]	301.1 [0.14]	317.0 [0.14]
15	A	Eu	T	*Haloterrigena turkmenica*	[3]	18	69.7 [2.10]	68.7 [0.26]	185.4 [12.7]	6.2 [0.06]	304.6 [0.27]	323.4 [0.28]
16	A	Eu	M	*Methanococcoides burtonii*	[5]	21	74.4 [1.20]	56.2 [0.06]	735.3 [21.7]	4.6 [0.01]	288.8 [0.09]	295.6 [0.08]
17	A	Eu	H	*Methanopyrus kandleri*	[6]	11	100.1 [9.08]	102.0 [0.71]	70.7 [2.0]	9.7 [0.05]	327.7 [0.33]	375.3 [0.34]
18	A	Eu	H	*Methanopyrus kandleri*	[6]	11	104.0 [10.60	105.5 [0.62]	73.7 [1.4]	9.8 [0.05]	329.3 [0.26]	379.0 [0.25]
19	A	Eu	M	*Natrialba asiatica*	[3]	15	77.1 [1.28]	65.2 [0.30]	195.0 [12.6]	5.9 [0.05]	300.7 [0.35]	316.7 [0.25]
20	A	Eu	T	*Natrinema pellirubrum*	[3]	18	69.5 [2.07]	69.0 [0.25]	194.4 [13.9]	6.1 [0.06]	304.8 [0.26]	323.7 [0.28]
21	A	Eu	H	*Pyrococcus horikoshii*	[7]	6	93.0 [8.75]	96.5 [0.57]	119.2 [11.8]	8.3 [0.14]	325.0 [0.30]	368.5 [0.45]
22	A	Eu	H	*Thermococcus peptonophilus*	[8]	6	95.6 [8.24]	85.7 [0.95]	59.1 [1.6]	9.1 [0.09]	318.6 [0.63]	357.0 [0.46]
23	A	Eu	H	*Thermococcus siculi*	[9]	6	83.8 [13.20]	88.6 [0.71]	205.3 [36.6]	7.1 [0.17]	320.5 [0.45]	358.2 [0.93]
24	B	Ab	T	*Acidimicrobium ferrooxidans*	[1]	21	67.4 [2.35]	67.6 [0.22]	196.4 [8.5]	6.1 [0.03]	303.4 [0.23]	320.9 [0.24]
25	B	Cy	T	*Synechococcus lividus*	[10]	11	74.2 [2.41]	76.2 [0.07]	288.1 [9.4]	6.2 [0.03]	311.6 [0.05]	338.2 [0.11]
26	B	Fi	M	*Listeria monocytogenes*	[11]	28	74.7 [1.34]	60.8 [0.37]	188.2 [23.8]	5.7 [0.09]	295.3 [0.50]	308.5 [0.34]
27	B	Fi	M	*Listeria monocytogenes*	[12]	28	74.2 [1.30]	60.1 [0.21]	185.4 [5.9]	5.7 [0.02]	294.3 [0.28]	307.2 [0.11]
28	B	Fi	M	*Listeria monocytogenes*	[12]	26	74.2 [1.30]	59.2 [0.39]	118.6 [6.5]	6.1 [0.05]	293.2 [0.55]	309.8 [0.23]
29	B	Fi	M	*Listeria monocytogenes*	[12]	27	74.4 [1.29]	61.1 [0.17]	238.1 [8.8]	5.5 [0.02]	295.7 [0.23]	307.8 [0.14]
30	B	Fi	M	*Listeria monocytogenes*	[12]	27	74.4 [1.31]	61.0 [0.19]	207.9 [6.4]	5.6 [0.02]	295.6 [0.25]	308.3 [0.13]
31	B	Fi	M	*Listeria monocytogenes*	[12]	27	74.3 [1.28]	60.9 [0.18]	203.4 [5.8]	5.7 [0.02]	295.4 [0.24]	308.1 [0.12]
32	B	Fi	M	*Streptococcus thermophilus*	[13]	23	77.3 [1.20]	63.7 [0.13]	299.1 [17.2]	5.5 [0.04]	299.0 [0.15]	312.7 [0.16]
33	B	Fi	M	*Streptococcus thermophilus*	[13]	24	75.7 [1.28]	62.7 [0.43]	134.7 [10.6]	6.2 [0.07]	297.8 [0.53]	314.3 [0.23]
34	B	Fi	M	*Streptococcus thermophilus*	[13]	22	79.8 [1.18]	64.9 [0.14]	222.5 [10.0]	5.8 [0.03]	300.4 [0.16]	315.9 [0.14]
35	B	Fi	M	*Streptococcus thermophilus*	[13]	18	78.6 [1.18]	64.6 [0.06]	484.2 [21.1]	5.2 [0.02]	300.0 [0.07]	314.4 [0.12]
36	B	Fi	M	*Streptococcus thermophilus*	[13]	22	80.4 [1.15]	65.6 [0.14]	199.5 [8.7]	5.9 [0.04]	301.2 [0.16]	317.7 [0.14]

(Table S1 continued.)

Code[a]	D.[b]	Taxa[c]	T.G.[d]	Strain name	Lit.[e]	Smp.[f]	$\Delta H_A^{\ddagger}$ [g]	ΔC_P [h]	n [i]	N_{ch} [j]	T_{mes} [k]	T_{opt} [l]
37	B	Fi	M	*Streptococcus thermophilus*	[13]	16	78.1 [1.25]	64.7 [0.09]	377.6 [23.5]	5.4 [0.04]	300.2 [0.11]	314.7 [0.16]
38	B	Fi	M	*Streptococcus thermophilus*	[13]	20	77.1 [1.22]	64.5 [0.08]	357.5 [19.4]	5.4 [0.03]	299.9 [0.10]	314.2 [0.13]
39	B	Fi	M	*Streptococcus thermophilus*	[13]	15	77.6 [1.25]	64.3 [0.09]	425.6 [26.8]	5.3 [0.04]	299.7 [0.11]	313.7 [0.16]
40	B	Fi	M	*Streptococcus thermophilus*	[13]	18	77.3 [1.26]	64.1 [0.10]	307.1 [13.8]	5.5 [0.03]	299.5 [0.12]	313.5 [0.13]
41	B	Fi	M	*Streptococcus thermophilus*	[13]	19	81.0 [1.21]	64.6 [0.05]	364.9 [7.5]	5.4 [0.01]	300.1 [0.06]	314.6 [0.08]
42	B	Fi	T	*Sulfobacillus thermosulfidooxidans*	[1]	20	68.5 [2.38]	68.2 [0.34]	139.5 [7.3]	6.5 [0.05]	304.0 [0.35]	323.0 [0.25]
43	B	Fi	T	*Thermaerobacter marianensis*	[14]	10	70.3 [2.60]	70.1 [1.51]	42.8 [2.5]	8.9 [0.11]	305.9 [1.63]	339.6 [0.48]
44	B	Ni	M	*Leptospirillum ferriphilum*	[1]	18	77.8 [1.21]	63.6 [0.11]	292.9 [14.1]	5.5 [0.03]	298.8 [0.14]	312.4 [0.14]
45	B	Ni	M	*Leptospirillum ferrooxidans*	[1]	16	75.4 [1.27]	62.6 [0.06]	520.5 [18.3]	5.1 [0.02]	297.7 [0.08]	309.9 [0.13]
46	B	Pr	M	*Acidithiobacillus caldus*	[1]	19	74.7 [1.40]	62.8 [0.33]	136.3 [5.6]	6.2 [0.03]	297.9 [0.42]	314.2 [0.19]
47	B	Pr	M	*Acidithiobacillus ferrooxidans*	[1]	17	71.2 [1.27]	50.5 [0.43]	96.3 [3.2]	5.9 [0.02]	278.9 [0.82]	299.7 [0.14]
48	B	Pr	M	*Acidithiobacillus thiooxidans*	[1]	20	72.2 [1.27]	60.1 [0.10]	491.7 [18.1]	5.0 [0.02]	294.4 [0.13]	304.0 [0.15]
49	B	Pr	M	*Aeromonas hydrophila*	[15]	30	74.4 [1.23]	58.0 [0.18]	183.7 [5.3]	5.6 [0.02]	291.5 [0.27]	303.9 [0.09]
50	B	Pr	M	*Desulfobacter curvatus*	[16]	24	73.6 [1.24]	53.6 [0.13]	301.2 [12.0]	5.0 [0.02]	284.5 [0.21]	293.8 [0.16]
51	B	Pr	P	*Desulfofaba gelida*	[17]	10	47.6 [6.56]	52.3 [0.16]	483.3 [87.9]	4.7 [0.09]	282.3 [0.29]	289.1 [0.59]
52	B	Pr	P	*Desulfofrigus fragile*	[17]	19	48.3 [6.32]	50.5 [1.32]	154.3 [16.1]	5.4 [0.07]	278.8 [2.55]	291.3 [0.43]
53	B	Pr	P	*Desulfofrigus marinus*	[17]	16	47.4 [10.12	45.6 [5.02]	383.3 [83.8]	4.5 [0.16]	267.2 [11.60]	285.3 [1.32]
54	B	Pr	M	*Desulforhopalus species*	[16]	29	71.4 [1.25]	60.6 [0.07]	620.3 [25.6]	4.9 [0.02]	295.0 [0.09]	305.1 [0.13]
55	B	Pr	P	*Desulfotalea arctica*	[17]	18	48.5 [5.73]	51.7 [0.40]	445.5 [29.9]	4.7 [0.02]	281.1 [0.72]	288.6 [0.11]
56	B	Pr	P	*Desulfotalea psychrophila*	[17]	39	50.4 [7.58]	45.2 [2.27]	330.9 [88.5]	4.5 [0.03]	267.3 [5.31]	285.3 [0.30]
57	B	Pr	M	*Erwinia amylovora*	[18]	10	74.8 [1.28]	58.5 [0.13]	381.0 [18.0]	5.1 [0.02]	292.1 [0.18]	301.1 [0.13]
58	B	Pr	M	*Erwinia amylovora*	[19]	11	74.4 [1.28]	58.9 [0.07]	729.1 [26.3]	4.7 [0.01]	292.8 [0.10]	301.3 [0.14]
59	B	Pr	M	*Escherichia coli*	[20]	26	75.6 [1.30]	63.1 [0.27]	223.2 [22.5]	5.7 [0.07]	298.3 [0.33]	312.1 [0.22]
60	B	Pr	M	*Escherichia coli*	[20]	27	76.3 [1.18]	63.8 [0.14]	261.0 [12.5]	5.6 [0.03]	299.1 [0.17]	313.0 [0.15]
61	B	Pr	M	*Escherichia coli*	[21]	12	75.4 [1.37]	62.1 [0.54]	225.5 [45.5]	5.7 [0.13]	297.1 [0.70]	310.1 [0.46]
62	B	Pr	M	*Escherichia coli*	[19]	22	75.8 [1.17]	64.6 [0.06]	623.3 [26.8]	5.1 [0.02]	300.1 [0.07]	314.6 [0.16]
63	B	Pr	M	*Escherichia coli*	[22]	20	78.1 [1.20]	63.9 [0.14]	242.1 [12.2]	5.7 [0.04]	299.2 [0.17]	313.5 [0.15]
64	B	Pr	M	*Escherichia coli*	[20]	25	76.6 [1.25]	63.6 [0.25]	183.7 [14.4]	5.9 [0.06]	298.9 [0.30]	313.9 [0.21]
65	B	Pr	M	*Escherichia coli*	[20]	48	75.9 [1.21]	62.9 [0.15]	231.3 [10.3]	5.7 [0.03]	298.0 [0.19]	311.5 [0.12]
66	B	Pr	M	*Escherichia coli*	[20]	27	75.5 [1.28]	63.4 [0.18]	235.7 [12.2]	5.7 [0.03]	298.7 [0.22]	312.5 [0.17]
67	B	Pr	M	*Escherichia coli*	[20]	25	76.4 [1.28]	63.9 [0.27]	199.1 [17.6]	5.8 [0.07]	299.2 [0.33]	314.0 [0.22]
68	B	Pr	M	*Escherichia coli*	[20]	57	74.2 [1.06]	63.5 [0.09]	304.1 [10.1]	5.5 [0.02]	298.7 [0.11]	311.9 [0.11]
69	B	Pr	M	*Escherichia coli*	[20]	26	75.5 [1.27]	63.3 [0.16]	255.2 [12.6]	5.6 [0.03]	298.5 [0.20]	312.0 [0.16]
70	B	Pr	M	*Escherichia coli*	[20]	26	75.4 [1.27]	63.0 [0.24]	217.3 [12.7]	5.7 [0.04]	298.1 [0.30]	311.9 [0.18]
71	B	Pr	M	*Escherichia coli*	[21]	12	75.7 [1.29]	61.0 [0.42]	127.6 [9.6]	6.2 [0.07]	295.6 [0.55]	311.9 [0.28]
72	B	Pr	M	*Escherichia coli*	[20]	26	75.4 [1.30]	63.2 [0.23]	208.1 [11.9]	5.8 [0.04]	298.3 [0.28]	312.4 [0.17]
73	B	Pr	M	*Escherichia coli*	[21]	10	75.5 [1.39]	61.9 [0.69]	183.7 [40.9]	5.8 [0.15]	296.7 [0.90]	310.6 [0.52]
74	B	Pr	M	*Escherichia coli*	[23]	20	74.6 [1.20]	64.5 [0.12]	404.7 [20.5]	5.3 [0.03]	299.9 [0.14]	314.0 [0.22]

(Table S1 continued.)

Code[a]	D.[b]	Taxa[c]	T.G.[d]	Strain name	Lit.[e]	Smp.[f]	$\Delta H_A^{\ddagger}$ [g]	ΔC_P [h]	n [i]	N_{ch} [j]	T_{mes} [k]	T_{opt} [l]
75	B	Pr	P	*Glaciecola punicea*	[24]	24	48.9 [6.41]	51.2 [0.92]	356.6 [52.3]	4.8 [0.05]	280.2 [1.74]	288.5 [0.17]
76	B	Pr	M	*Klebsiella oxytoca*	[25]	27	74.4 [1.32]	61.5 [0.31]	174.2 [11.2]	5.8 [0.05]	296.2 [0.41]	310.1 [0.17]
77	B	Pr	M	*Paracoccus halodenitrificans*	[21]	13	74.8 [1.28]	59.5 [0.29]	173.6 [12.7]	5.7 [0.06]	293.5 [0.40]	306.7 [0.34]
78	B	Pr	M	*Paracoccus halodenitrificans*	[21]	13	79.9 [1.15]	61.5 [0.04]	837.7 [31.7]	4.8 [0.02]	296.3 [0.05]	307.7 [0.09]
79	B	Pr	M	*Paracoccus halodenitrificans*	[21]	13	79.7 [1.17]	61.4 [0.04]	770.0 [29.8]	4.8 [0.02]	296.1 [0.06]	307.3 [0.10]
80	B	Pr	M	*Paracoccus halodenitrificans*	[21]	11	80.4 [1.19]	61.2 [0.05]	699.7 [38.0]	4.9 [0.03]	295.8 [0.07]	306.7 [0.10]
81	B	Pr	M	*Pseudomonas aeruginosa*	[22]	10	75.5 [1.34]	62.7 [0.37]	240.3 [19.4]	5.6 [0.06]	297.8 [0.48]	311.0 [0.31]
82	B	Pr	M	*Pseudomonas fluorescens*	[22]	16	73.4 [1.26]	56.0 [0.27]	137.6 [5.0]	5.8 [0.03]	288.4 [0.42]	303.2 [0.22]
83	B	Pr	M	*Pseudomonas fluorescens*	[26]	52	73.2 [1.20]	55.8 [0.16]	185.9 [3.8]	5.5 [0.01]	288.1 [0.24]	300.1 [0.06]
84	B	Pr	M	*Pseudomonas putida*	[26]	85	72.9 [1.13]	55.3 [0.16]	158.1 [2.2]	5.6 [0.01]	287.4 [0.25]	300.8 [0.06]
85	B	Pr	P	*Psychrobacter glacincola*	[27]	13	46.4 [6.36]	47.8 [1.45]	405.9 [80.0]	4.5 [0.04]	273.4 [3.11]	285.9 [0.27]
86	B	Pr	P	*Shewanella gelidimarina*	[28]	26	50.7 [5.77]	52.0 [0.48]	502.1 [53.2]	4.6 [0.04]	281.8 [0.85]	289.0 [0.11]
87	B	Pr	P	*Shewanella gelidimarina*	[28]	28	48.9 [6.04]	50.7 [0.80]	408.4 [52.0]	4.7 [0.03]	279.3 [1.52]	287.8 [0.17]
88	B	Pr	P	*Shewanella gelidimarina*	[28]	28	48.9 [6.00]	50.7 [0.79]	408.6 [51.6]	4.7 [0.03]	279.3 [1.49]	287.8 [0.16]
89	B	Pr	M	*Xanthomonas campestris*	[19]	8	73.2 [1.32]	59.8 [0.11]	611.5 [33.8]	4.9 [0.02]	294.0 [0.16]	303.4 [0.23]
90	B	Pr	M	*Xylella fastidiosa*	[19]	9	77.1 [1.25]	59.3 [0.08]	566.4 [30.6]	4.9 [0.03]	293.3 [0.11]	302.3 [0.15]
91	E	Ac	M	*Aleuroglyphus ovatus*	[29]	10	74.1 [1.28]	56.1 [0.20]	258.5 [9.3]	5.2 [0.02]	288.6 [0.31]	298.5 [0.13]
92	E	Ac	M	*Diaphorina citri*	[30]	5	75.7 [1.32]	58.8 [0.30]	553.6 [50.5]	4.9 [0.05]	292.6 [0.40]	301.2 [0.65]
93	E	Ac	M	*Euseius finlandicus*	[31]	6	74.7 [1.34]	59.1 [0.35]	379.8 [52.8]	5.1 [0.08]	293.0 [0.50]	302.4 [0.43]
94	E	Ac	M	*Phyllocoptruta oleivora*	[32]	6	75.0 [1.33]	58.9 [0.37]	432.0 [49.5]	5.0 [0.06]	292.7 [0.52]	301.7 [0.74]
95	E	Ac	M	*Stethorus punctillum*	[33]	6	77.0 [1.29]	61.7 [0.39]	419.7 [50.7]	5.2 [0.08]	296.4 [0.50]	307.8 [0.87]
96	E	Ac	M	*Tetranychus evansi*	[34]	5	75.3 [1.33]	60.2 [0.31]	373.1 [50.0]	5.2 [0.08]	294.6 [0.43]	304.7 [0.42]
97	E	Ac	M	*Tetranychus evansi*	[34]	5	75.5 [1.36]	60.4 [0.36]	353.0 [48.6]	5.2 [0.08]	294.8 [0.50]	305.2 [0.45]
98	E	Ac	M	*Tetranychus evansi*	[34]	5	75.5 [1.33]	60.4 [0.35]	391.4 [52.2]	5.2 [0.08]	294.8 [0.48]	305.1 [0.44]
99	E	Ac	M	*Tetranychus evansi*	[34]	5	75.4 [1.30]	60.5 [0.31]	363.3 [52.4]	5.2 [0.08]	294.9 [0.42]	305.3 [0.43]
100	E	Ac	M	*Tetranychus evansi*	[34]	5	75.3 [1.32]	60.4 [0.37]	350.9 [50.7]	5.2 [0.08]	294.8 [0.51]	305.2 [0.49]
101	E	Ac	M	*Tetranychus evansi*	[34]	5	75.4 [1.30]	60.5 [0.31]	361.9 [53.0]	5.2 [0.08]	295.0 [0.42]	305.4 [0.47]
102	E	Ac	M	*Tetranychus evansi*	[34]	5	75.4 [1.34]	60.4 [0.41]	370.2 [53.8]	5.2 [0.10]	294.7 [0.58]	305.1 [0.59]
103	E	Ac	M	*Tyrophagus putrescentiae*	[29]	10	73.8 [1.33]	56.0 [0.37]	190.9 [10.2]	5.5 [0.03]	288.5 [0.60]	300.3 [0.18]
104	E	Ac	M	*Tyrophagus putrescentiae*	[35]	6	75.2 [1.30]	59.2 [0.10]	604.1 [34.1]	4.9 [0.02]	293.2 [0.14]	302.1 [0.18]
105	E	As	F	*Brettanomyces bruxellensis*	[36]	6	41.5 [1.00]	63.0 [0.27]	328.3 [14.0]	5.4 [0.03]	298.2 [0.33]	309.2 [0.59]
106	E	As	F	*Candida valida*	[37]	15	41.9 [0.94]	62.2 [0.05]	434.6 [10.3]	5.2 [0.01]	297.1 [0.06]	307.4 [0.10]
107	E	As	F	*Hanseniaspora uvarum*	[38]	21	40.6 [0.98]	59.0 [0.06]	416.6 [7.4]	5.0 [0.01]	292.9 [0.08]	300.1 [0.07]
108	E	As	F	*Kluyveromyces marxianus*	[38]	27	39.5 [0.83]	64.0 [0.10]	233.6 [11.5]	5.7 [0.04]	299.4 [0.12]	311.3 [0.15]
109	E	As	F	*Monascus ruber*	[39]	5	41.1 [1.02]	63.1 [0.12]	333.3 [13.9]	5.4 [0.03]	298.3 [0.15]	309.4 [0.25]
110	E	As	F	*Monascus ruber*	[39]	5	41.0 [0.98]	63.8 [0.16]	327.2 [15.0]	5.4 [0.03]	299.1 [0.19]	310.9 [0.34]
111	E	As	F	*Monascus ruber*	[39]	5	41.2 [1.02]	63.3 [0.13]	329.6 [13.8]	5.4 [0.03]	298.5 [0.16]	309.8 [0.27]
112	E	As	F	*Monascus ruber*	[39]	5	42.1 [1.02]	63.0 [0.10]	343.8 [13.7]	5.4 [0.03]	298.1 [0.13]	309.1 [0.21]

(Table S1 continued.)

Code[a]	D.[b]	Taxa[c]	T.G.[d]	Strain name	Lit.[e]	Smp.[f]	$\Delta H_A^{\ddagger}$ [g]	ΔC_P [h]	n [i]	N_{ch} [j]	T_{mes} [k]	T_{opt} [l]
113	E	As	F	*Monascus ruber*	[39]	5	41.7 [1.01]	64.3 [0.20]	332.6 [14.8]	5.4 [0.03]	299.7 [0.24]	312.2 [0.45]
114	E	As	F	*Monascus ruber*	[39]	5	42.0 [1.01]	64.7 [0.21]	335.6 [13.9]	5.5 [0.03]	300.1 [0.24]	313.0 [0.46]
115	E	As	F	*Monascus ruber*	[39]	5	41.8 [1.02]	62.6 [0.09]	340.1 [12.4]	5.3 [0.02]	297.6 [0.11]	308.1 [0.17]
116	E	As	F	*Monascus ruber*	[39]	5	41.3 [1.01]	64.1 [0.18]	332.6 [15.0]	5.4 [0.03]	299.4 [0.21]	311.6 [0.38]
117	E	As	F	*Monascus ruber*	[39]	5	41.4 [1.01]	64.4 [0.20]	330.0 [15.3]	5.5 [0.03]	299.8 [0.24]	312.2 [0.45]
118	E	As	F	*Monascus ruber*	[39]	5	42.1 [1.02]	62.7 [0.09]	347.7 [12.7]	5.3 [0.02]	297.8 [0.11]	308.5 [0.18]
119	E	As	F	*Monascus ruber*	[39]	5	41.6 [1.02]	64.6 [0.20]	332.4 [15.3]	5.5 [0.03]	300.0 [0.23]	312.8 [0.44]
120	E	As	F	*Pichia fermentans*	[38]	21	36.3 [1.02]	59.2 [0.10]	257.2 [5.1]	5.4 [0.01]	293.2 [0.14]	300.8 [0.08]
121	E	As	F	*Saccharomyces arboricolus*	[38]	24	40.2 [0.92]	60.1 [0.06]	373.2 [7.5]	5.2 [0.01]	294.4 [0.08]	302.5 [0.08]
122	E	As	F	*Saccharomyces bayanus*	[38]	21	39.5 [0.99]	59.5 [0.07]	333.2 [7.1]	5.2 [0.01]	293.6 [0.10]	301.2 [0.09]
123	E	As	F	*Saccharomyces bayanus*	[38]	24	39.9 [0.97]	59.3 [0.07]	342.4 [5.5]	5.2 [0.01]	293.3 [0.09]	300.9 [0.07]
124	E	As	F	*Saccharomyces cariocanus*	[38]	27	36.8 [0.91]	60.9 [0.06]	383.6 [7.8]	5.2 [0.01]	295.5 [0.08]	304.1 [0.09]
125	E	As	F	*Saccharomyces cerevisiae*	[38]	21	38.2 [0.88]	62.5 [0.06]	343.7 [10.0]	5.3 [0.02]	297.5 [0.08]	307.7 [0.11]
126	E	As	F	*Saccharomyces cerevisiae*	[38]	27	37.7 [0.94]	62.4 [0.07]	313.0 [6.8]	5.4 [0.01]	297.4 [0.08]	307.5 [0.09]
127	E	As	F	*Saccharomyces cerevisiae*	[38]	30	39.5 [0.89]	61.9 [0.05]	360.8 [6.8]	5.3 [0.01]	296.8 [0.07]	306.6 [0.09]
128	E	As	F	*Saccharomyces cerevisiae*	[38]	30	39.9 [0.87]	61.9 [0.06]	337.6 [6.6]	5.3 [0.01]	296.8 [0.08]	306.6 [0.09]
129	E	As	F	*Saccharomyces cerevisiae*	[38]	30	36.9 [0.86]	62.8 [0.06]	302.0 [7.3]	5.4 [0.02]	297.9 [0.08]	308.4 [0.09]
130	E	As	F	*Saccharomyces cerevisiae*	[38]	24	39.5 [0.87]	62.4 [0.06]	353.6 [8.6]	5.3 [0.01]	297.3 [0.07]	307.5 [0.10]
131	E	As	F	*Saccharomyces cerevisiae*	[38]	24	39.8 [0.85]	62.4 [0.05]	358.2 [8.6]	5.3 [0.01]	297.4 [0.07]	307.8 [0.10]
132	E	As	F	*Saccharomyces cerevisiae*	[38]	24	37.9 [0.91]	62.4 [0.08]	301.6 [8.1]	5.4 [0.02]	297.4 [0.10]	307.5 [0.11]
133	E	As	F	*Saccharomyces cerevisiae*	[38]	24	37.5 [0.95]	62.3 [0.10]	245.9 [7.5]	5.6 [0.02]	297.2 [0.13]	307.2 [0.12]
134	E	As	F	*Saccharomyces cerevisiae*	[38]	27	40.8 [0.86]	62.2 [0.05]	371.2 [7.9]	5.3 [0.01]	297.1 [0.07]	307.2 [0.09]
135	E	As	F	*Saccharomyces cerevisiae*	[40]	7	39.5 [1.04]	62.3 [0.11]	332.0 [14.1]	5.3 [0.03]	297.3 [0.14]	307.4 [0.22]
136	E	As	F	*Saccharomyces kudriavzevii*	[38]	21	40.2 [0.98]	58.3 [0.06]	392.4 [6.3]	5.0 [0.01]	291.9 [0.08]	298.6 [0.07]
137	E	As	F	*Saccharomyces kudriavzevii*	[38]	21	39.6 [0.97]	58.6 [0.06]	401.1 [7.2]	5.0 [0.01]	292.3 [0.09]	299.2 [0.07]
138	E	As	F	*Saccharomyces kudriavzevii*	[38]	21	40.5 [0.99]	58.4 [0.06]	417.4 [7.5]	5.0 [0.01]	292.0 [0.08]	298.8 [0.07]
139	E	As	F	*Saccharomyces kudriavzevii*	[38]	21	39.8 [0.99]	58.6 [0.06]	373.3 [6.3]	5.1 [0.01]	292.3 [0.09]	299.2 [0.07]
140	E	As	F	*Saccharomyces mikatae*	[38]	24	35.7 [0.98]	60.8 [0.08]	318.9 [7.2]	5.3 [0.01]	295.3 [0.10]	303.7 [0.10]
141	E	As	F	*Saccharomyces paradoxus*	[38]	24	38.2 [0.93]	61.0 [0.07]	333.4 [6.8]	5.3 [0.01]	295.6 [0.09]	304.4 [0.09]
142	E	As	F	*Saccharomyces paradoxus*	[38]	27	38.8 [0.95]	61.1 [0.07]	319.5 [6.1]	5.3 [0.01]	295.7 [0.09]	304.6 [0.09]
143	E	As	F	*Saccharomyces paradoxus*	[38]	27	38.0 [0.91]	61.4 [0.07]	326.8 [6.9]	5.3 [0.01]	296.1 [0.09]	305.1 [0.09]
144	E	As	F	*Saccharomyces rouxii*	[41]	4	39.2 [1.10]	60.1 [0.25]	339.7 [13.9]	5.2 [0.03]	294.3 [0.34]	302.4 [0.51]
145	E	As	F	*Saccharomyces rouxii*	[41]	6	39.5 [1.04]	61.1 [0.10]	334.8 [11.2]	5.3 [0.02]	295.8 [0.13]	304.8 [0.18]
146	E	As	F	*Saccharomyces rouxii*	[41]	5	39.5 [1.06]	61.6 [0.11]	323.8 [12.5]	5.3 [0.02]	296.4 [0.15]	305.8 [0.22]
147	E	As	F	*Torulaspora delbrueckii*	[38]	21	39.1 [0.95]	60.3 [0.07]	331.4 [6.8]	5.3 [0.01]	294.7 [0.10]	302.9 [0.10]
148	E	Co	M	*Paronychiurus kimi*	[42]	5	74.6 [1.28]	56.9 [0.15]	630.6 [44.2]	4.7 [0.03]	289.9 [0.23]	297.2 [0.21]
149	E	In	M	*Acyrthosiphon kondoi*	[43]	8	73.8 [1.30]	56.2 [0.41]	411.1 [54.0]	4.9 [0.09]	288.7 [0.63]	296.6 [1.00]
150	E	In	M	*Acyrthosiphon pisum*	[44]	10	72.9 [1.28]	54.6 [0.38]	286.4 [30.7]	5.1 [0.05]	286.3 [0.63]	295.5 [0.22]

(Table S1 continued.)

Code[a]	D.[b]	Taxa[c]	T.G.[d]	Strain name	Lit.[e]	Smp.[f]	$\Delta H_A^{\ddagger}$ [g]	ΔC_P [h]	n [i]	N_{ch} [j]	T_{mes} [k]	T_{opt} [l]
151	E	In	M	*Adoxophyes orana*	[45]	7	74.8 [1.26]	57.2 [0.09]	742.0 [32.7]	4.7 [0.02]	290.2 [0.13]	297.5 [0.14]
152	E	In	M	*Amblyseius womersleyi*	[46]	8	76.3 [1.28]	61.8 [0.24]	383.8 [43.9]	5.2 [0.07]	296.6 [0.32]	308.1 [0.34]
153	E	In	M	*Amitus fuscipennis*	[47]	12	74.8 [1.32]	57.3 [0.13]	520.0 [30.7]	4.8 [0.03]	290.4 [0.20]	298.1 [0.15]
154	E	In	M	*Aphis citricola*	[48]	6	74.8 [1.28]	57.5 [0.06]	1143.6 [48.5]	4.5 [0.01]	290.7 [0.08]	298.4 [0.13]
155	E	In	M	*Aphis gossypii*	[49]	5	74.5 [1.29]	57.9 [0.19]	460.6 [43.0]	4.9 [0.04]	291.3 [0.28]	299.5 [0.23]
156	E	In	M	*Aphis gossypii*	[50]	5	74.1 [1.27]	57.3 [0.25]	543.0 [51.7]	4.8 [0.04]	290.5 [0.38]	298.1 [0.28]
157	E	In	M	*Aphis gossypii*	[51]	5	73.7 [1.35]	57.0 [0.40]	257.0 [46.5]	5.3 [0.10]	290.0 [0.61]	300.0 [0.45]
158	E	In	M	*Aphis gossypii*	[52]	6	75.1 [1.26]	58.6 [0.10]	504.4 [18.9]	4.9 [0.02]	292.3 [0.15]	300.8 [0.15]
159	E	In	M	*Aphis gossypii*	[48]	5	73.9 [1.30]	55.8 [0.26]	401.1 [26.1]	4.9 [0.03]	288.2 [0.42]	296.1 [0.20]
160	E	In	M	*Aphis gossypii*	[53]	8	74.4 [1.28]	57.8 [0.13]	545.6 [27.4]	4.8 [0.02]	291.2 [0.18]	299.1 [0.17]
161	E	In	M	*Aphis nasturti*	[54]	5	73.8 [1.35]	57.5 [0.41]	374.5 [53.2]	5.0 [0.07]	290.8 [0.61]	299.4 [0.41]
162	E	In	M	*Aphis punicae*	[55]	5	74.8 [1.37]	58.8 [0.51]	461.4 [52.8]	5.0 [0.06]	292.6 [0.71]	301.4 [1.04]
163	E	In	M	*Aphis spiraecola*	[56]	7	73.9 [1.28]	58.0 [0.09]	780.9 [40.0]	4.7 [0.02]	291.5 [0.13]	299.3 [0.18]
164	E	In	M	*Aulacorthum solani*	[57]	4	74.2 [1.29]	56.6 [0.24]	489.9 [41.1]	4.8 [0.04]	289.4 [0.36]	297.0 [0.42]
165	E	In	M	*Bactrocera dorsalis*	[58]	7	74.7 [1.31]	60.2 [0.07]	1015.3 [66.4]	4.7 [0.02]	294.5 [0.10]	304.4 [0.20]
166	E	In	M	*Bemisia argentifolii*	[59]	5	74.8 [1.31]	58.3 [0.21]	385.0 [24.1]	5.1 [0.03]	291.9 [0.30]	300.8 [0.25]
167	E	In	M	*Bemisia argentifolii*	[60]	5	75.4 [1.32]	59.6 [0.22]	467.7 [41.7]	5.0 [0.04]	293.7 [0.30]	303.1 [0.34]
168	E	In	M	*Brevicoryne brassicae*	[61]	4	73.9 [1.28]	55.4 [0.16]	570.2 [40.2]	4.7 [0.03]	287.6 [0.25]	294.6 [0.17]
169	E	In	M	*Calandra oryzae*	[62]	7	79.1 [1.23]	59.5 [0.04]	1051.4 [46.1]	4.6 [0.02]	293.5 [0.05]	302.9 [0.09]
170	E	In	M	*Callosobruchus maculatus*	[63]	7	76.6 [1.27]	62.0 [0.07]	576.2 [31.6]	5.0 [0.03]	296.9 [0.09]	308.6 [0.16]
171	E	In	M	*Clavigralla tomentosicollis*	[64]	6	76.5 [1.35]	61.3 [0.37]	347.6 [42.7]	5.3 [0.08]	296.0 [0.49]	307.3 [0.60]
172	E	In	M	*Cryptolestes ferrugineus*	[65]	9	78.4 [1.25]	62.0 [0.06]	458.8 [18.7]	5.1 [0.02]	296.9 [0.08]	308.5 [0.11]
173	E	In	M	*Dactylopius austrinus*	[66]	8	77.0 [1.25]	60.1 [0.07]	653.2 [40.3]	4.9 [0.03]	294.4 [0.10]	304.2 [0.13]
174	E	In	M	*Elasmopalpus lignosellus*	[67]	9	75.6 [1.25]	60.2 [0.10]	516.8 [30.1]	5.0 [0.03]	294.6 [0.14]	304.4 [0.18]
175	E	In	M	*Eriosoma lanigerum*	[68]	6	73.7 [1.30]	56.7 [0.26]	346.8 [33.0]	5.0 [0.05]	289.5 [0.39]	298.0 [0.21]
176	E	In	M	*Frankliniella occidentalis*	[69]	6	77.4 [1.24]	62.9 [0.36]	351.2 [40.7]	5.3 [0.08]	298.1 [0.44]	310.8 [0.80]
177	E	In	M	*Hypothenemus hampei*	[70]	5	75.3 [1.31]	58.4 [0.13]	582.7 [45.9]	4.8 [0.04]	292.1 [0.19]	300.4 [0.22]
178	E	In	M	*Iphiseius degenerans*	[71]	5	74.3 [1.34]	58.2 [0.33]	347.9 [38.9]	5.1 [0.06]	291.7 [0.48]	300.8 [0.32]
179	E	In	M	*Liposcelis bostrychophila*	[72]	7	75.6 [1.31]	60.2 [0.21]	414.1 [37.2]	5.1 [0.05]	294.5 [0.29]	304.5 [0.26]
180	E	In	M	*Liposcelis entomophila*	[73]	7	75.9 [1.31]	60.0 [0.18]	446.8 [32.5]	5.1 [0.04]	294.3 [0.25]	304.1 [0.23]
181	E	In	M	*Macrolophus pygmaeus*	[74]	5	74.5 [1.32]	57.8 [0.22]	465.2 [41.0]	4.9 [0.04]	291.1 [0.32]	299.3 [0.25]
182	E	In	M	*Macrolophus pygmaeus*	[74]	5	74.6 [1.30]	57.8 [0.23]	530.6 [44.6]	4.9 [0.04]	291.2 [0.34]	299.2 [0.28]
183	E	In	M	*Muscidifurax raptor*	[75]	9	75.4 [1.32]	59.2 [0.25]	398.5 [39.3]	5.1 [0.06]	293.2 [0.36]	302.6 [0.25]
184	E	In	M	*Muscidifurax raptorellus*	[76]	10	77.5 [1.27]	59.9 [0.11]	426.5 [29.4]	5.1 [0.04]	294.1 [0.14]	303.9 [0.20]
185	E	In	M	*Muscidifurax zaraptor*	[77]	7	76.6 [1.31]	60.2 [0.27]	413.7 [41.9]	5.1 [0.07]	294.5 [0.37]	304.6 [0.51]
186	E	In	M	*Myzus persicae*	[61]	5	74.2 [1.30]	56.9 [0.17]	599.1 [37.5]	4.7 [0.03]	289.8 [0.25]	297.2 [0.34]
187	E	In	M	*Myzus persicae*	[78]	5	73.4 [1.31]	55.9 [0.34]	266.1 [26.8]	5.2 [0.05]	288.2 [0.53]	297.9 [0.23]
188	E	In	M	*Nephaspis oculatus*	[79]	5	75.3 [1.32]	58.8 [0.18]	524.1 [44.1]	4.9 [0.04]	292.6 [0.26]	301.2 [0.27]

(Table S1 continued.)

Code[a]	D.[b]	Taxa[c]	T.G.[d]	Strain name	Lit.[e]	Smp.[f]	$\Delta H_A^{\ddagger}$ [g]	ΔC_P [h]	n [i]	N_{ch} [j]	T_{mes} [k]	T_{opt} [l]
189	E	In	M	*Nephus bisignatus*	[80]	5	75.2 [1.28]	58.4 [0.14]	583.9 [32.6]	4.8 [0.02]	292.1 [0.21]	300.3 [0.22]
190	E	In	M	*Nephus includens*	[80]	6	76.0 [1.30]	59.5 [0.07]	639.7 [26.6]	4.8 [0.02]	293.6 [0.10]	302.7 [0.14]
191	E	In	M	*Oryzaephilus surinamensis*	[81]	10	74.7 [1.38]	59.4 [0.44]	388.2 [46.9]	5.1 [0.08]	293.5 [0.63]	303.0 [0.39]
192	E	In	M	*Oryzaephilus surinamensis*	[81]	10	74.8 [1.38]	59.6 [0.39]	376.5 [47.6]	5.1 [0.08]	293.7 [0.56]	303.5 [0.36]
193	E	In	M	*Oryzaephilus surinamensis*	[81]	10	74.6 [1.37]	59.4 [0.31]	375.4 [40.0]	5.1 [0.06]	293.4 [0.43]	303.0 [0.35]
194	E	In	M	*Oryzaephilus surinamensis*	[81]	9	74.7 [1.36]	59.2 [0.27]	414.6 [37.0]	5.1 [0.05]	293.2 [0.38]	302.4 [0.31]
195	E	In	M	*Oryzaephilus surinamensis*	[82]	6	74.7 [1.35]	59.0 [0.31]	383.9 [33.5]	5.1 [0.04]	292.8 [0.44]	302.1 [0.35]
196	E	In	M	*Oryzaephilus surinamensis*	[82]	6	74.7 [1.41]	59.4 [0.32]	376.0 [42.3]	5.1 [0.06]	293.4 [0.45]	302.9 [0.37]
197	E	In	M	*Oryzaephilus surinamensis*	[82]	6	74.9 [1.36]	59.8 [0.51]	373.4 [47.5]	5.2 [0.09]	294.0 [0.76]	303.8 [0.42]
198	E	In	M	*Plutella xylostella*	[83]	10	74.7 [1.30]	58.7 [0.12]	479.9 [25.4]	4.9 [0.03]	292.4 [0.17]	301.0 [0.15]
199	E	In	M	*Propylea dissecta*	[84]	5	74.3 [1.39]	58.8 [0.52]	253.2 [53.6]	5.4 [0.10]	292.6 [0.75]	303.2 [0.48]
200	E	In	M	*Pterohelaeus darlingensis*	[85]	4	75.4 [1.32]	59.1 [0.12]	607.6 [42.5]	4.8 [0.03]	293.0 [0.16]	301.8 [0.21]
201	E	In	M	*Rhyzopertha dominica*	[81]	13	73.4 [1.31]	60.2 [0.18]	438.1 [28.6]	5.1 [0.03]	294.5 [0.24]	304.3 [0.27]
202	E	In	M	*Rhyzopertha dominica*	[81]	12	73.2 [1.37]	57.4 [0.47]	214.0 [19.2]	5.4 [0.05]	290.6 [0.70]	301.7 [0.29]
203	E	In	M	*Rhyzopertha dominica*	[81]	14	73.3 [1.36]	60.5 [0.20]	437.2 [31.6]	5.1 [0.04]	294.9 [0.27]	305.0 [0.28]
204	E	In	M	*Rhyzopertha dominica*	[81]	14	73.4 [1.40]	59.7 [0.33]	326.7 [26.7]	5.2 [0.04]	293.8 [0.46]	303.8 [0.35]
205	E	In	M	*Saccharicoccus sacchari*	[86]	5	76.2 [1.32]	60.0 [0.19]	583.4 [54.4]	4.9 [0.05]	294.3 [0.25]	304.0 [0.38]
206	E	In	M	*Scolothrips longicornis*	[87]	6	75.7 [1.28]	60.8 [0.11]	617.2 [42.7]	4.9 [0.03]	295.4 [0.14]	305.8 [0.23]
207	E	In	M	*Sitobion miscanthi*	[88]	9	75.0 [1.26]	56.5 [0.07]	740.3 [29.7]	4.6 [0.02]	289.2 [0.11]	296.2 [0.10]
208	E	In	M	*Sitophilus oryzae*	[81]	10	73.4 [1.37]	56.5 [0.54]	235.3 [27.9]	5.3 [0.07]	289.2 [0.84]	299.5 [0.31]
209	E	In	M	*Sitophilus oryzae*	[81]	10	73.2 [1.39]	56.5 [0.56]	212.5 [33.1]	5.4 [0.08]	289.2 [0.88]	300.2 [0.37]
210	E	In	M	*Sitophilus oryzae*	[81]	10	73.4 [1.36]	56.5 [0.49]	257.8 [27.2]	5.2 [0.06]	289.2 [0.77]	299.0 [0.28]
211	E	In	M	*Sitophilus oryzae*	[89]	7	73.4 [1.36]	56.4 [0.44]	265.3 [27.1]	5.2 [0.06]	289.0 [0.68]	298.7 [0.26]
212	E	In	M	*Sitophilus oryzae*	[89]	7	73.3 [1.35]	56.3 [0.51]	228.9 [29.5]	5.3 [0.07]	289.0 [0.79]	299.5 [0.32]
213	E	In	M	*Sitophilus oryzae*	[89]	7	73.2 [1.33]	56.4 [0.58]	205.8 [25.6]	5.4 [0.08]	289.1 [0.91]	300.4 [0.37]
214	E	In	M	*Tetranychus mcdanieli*	[33]	11	77.2 [1.18]	61.1 [0.04]	1264.1 [59.0]	4.6 [0.02]	295.7 [0.05]	306.9 [0.11]
215	E	In	M	*Thrips tabaci*	[90]	5	75.1 [1.25]	57.1 [0.08]	858.9 [36.4]	4.6 [0.01]	290.2 [0.12]	297.4 [0.14]
216	E	In	M	*Toxoptera aurantii*	[91]	7	74.1 [1.29]	58.0 [0.10]	746.5 [38.8]	4.7 [0.02]	291.4 [0.14]	299.2 [0.18]
217	E	In	M	*Toxoptera citricida*	[92]	7	74.8 [1.30]	58.4 [0.07]	782.4 [39.3]	4.7 [0.02]	292.1 [0.11]	300.2 [0.15]
218	E	In	M	*Toxoptera citricidus*	[48]	11	75.2 [1.26]	57.9 [0.06]	803.1 [40.3]	4.7 [0.02]	291.3 [0.10]	299.0 [0.11]
219	E	In	M	*Tribolium castaneum*	[93]	8	76.6 [1.34]	62.3 [0.38]	442.2 [62.9]	5.2 [0.10]	297.3 [0.49]	309.3 [0.63]
220	E	In	M	*Tribolium castaneum*	[81]	13	74.7 [1.36]	60.1 [0.36]	259.9 [21.5]	5.4 [0.05]	294.4 [0.50]	305.5 [0.26]
221	E	In	M	*Tribolium castaneum*	[81]	12	74.6 [1.42]	59.4 [0.53]	217.2 [22.1]	5.5 [0.07]	293.4 [0.78]	305.0 [0.27]
222	E	In	M	*Tribolium castaneum*	[81]	9	74.5 [1.42]	58.8 [0.59]	192.2 [24.3]	5.6 [0.08]	292.6 [0.86]	304.8 [0.35]
223	E	In	M	*Trichogramma pretiosum*	[94]	6	75.6 [1.34]	60.4 [0.37]	437.7 [57.0]	5.1 [0.07]	294.8 [0.49]	304.9 [0.76]
224	E	In	M	*Trichogramma pretiosum*	[94]	6	75.4 [1.32]	59.5 [0.25]	444.8 [47.5]	5.0 [0.06]	293.6 [0.34]	302.9 [0.42]
225	E	In	M	*Trichogrammatoidea bactrae*	[95]	6	75.6 [1.27]	58.5 [0.06]	880.1 [40.3]	4.6 [0.02]	292.2 [0.09]	300.6 [0.13]
226	E	In	M	*Trichogrammatoidea bactrae*	[96]	6	74.8 [1.31]	58.4 [0.22]	388.0 [33.7]	5.1 [0.04]	292.0 [0.31]	300.8 [0.23]

(Table S1 continued.)

Code[a]	D.[b]	Taxa[c]	T.G.[d]	Strain name	Lit.[e]	Smp.[f]	$\Delta H_A^{\ddagger}$ [g]	ΔC_P [h]	n [i]	N_{ch} [j]	T_{mes} [k]	T_{opt} [l]
227	E	In	M	*Trichomalopsis sarcophagae*	[97]	7	76.2 [1.25]	59.1 [0.05]	1267.4 [75.7]	4.5 [0.02]	293.0 [0.07]	302.0 [0.15]
228	E	In	M	*Urolepis rufipes*	[98]	8	76.4 [1.26]	60.2 [0.10]	544.0 [41.6]	5.0 [0.04]	294.6 [0.13]	304.4 [0.18]
229	E	Pl	M	*Chlorella*	[99]	6	75.8 [1.33]	60.9 [0.43]	204.6 [16.8]	5.7 [0.06]	295.4 [0.60]	308.2 [0.29]
230	E	Pl	M	*Chlorella pyrenoidosa*	[99]	9	77.4 [1.27]	63.1 [0.05]	692.3 [35.4]	5.0 [0.02]	298.3 [0.06]	311.3 [0.13]

[a] Strain code.
[b] Domain: A=Archaea; B=bacteria; E=Eukarya.
[c] Taxa: Cr=Crenarchaeota; Eu=Euryarchaeota; Ab=Actinobacteria; Cy=Cyanobacteria; Fi=Firmicutes; Ni=Nitrospirae; Pr=Proteobacteria; Ac=Acari; As=Ascomycota; Co=Collembola; In=Insecta; Pl=Plantae.
[d] Thermal group: P=psychrophile; M=mesophile; F= Ascomycota; T=thermophile; H=hyperthermophile.
[e] Source of data.
[f] Sample size.
[g] Enthalpy of activation (J/mol).
[h] Heat capacity change (J/K mol-amino acid-residue).
[i] Number of amino acid residues.
[j] Average number of non-polar hydrogen atoms per amino acid residue.
[k] Temperature of minimal denaturation (K).
[l] Temperature of optimal growth (K).

References

[1] Franzmann PD, Haddad CM, Hawkes RB, Robertson WJ, Plumb JJ (2005) Effects of temperature on the rates of iron and sulfur oxidation by selected bioleaching Bacteria and Archaea: Application of the Ratkowsky equation. Miner Eng 18: 1304–1314.

[2] Kashefi K, Lovley DR (2003) Extending the upper temperature limit for life. Science 301: 934.

[3] Robinson JL, Pyzyna B, Atrasz RG, Henderson CA, Morrill KL, et al. (2005) Growth kinetics of extremely halophilic Archaea (family Halobacteriaceae) as revealed by Arrhenius plots. J Bacteriol 187: 923–929.

[4] McMeekin TA, Franzmann PD (1988) Effect of temperature on the growth rates of halotolerant and halophilic bacteria isolated from Antarctic saline lakes. Polar Biol 8: 281–285.

[5] Franzmann PD, Springer N, Ludwig W, Conway de Macario E, Rohde M (1992) A methanogenic Archaeon from Ace Lake, Antarctica: *Methanococcoides burtonii* sp. nov. Syst Appl Microbiol 15: 573–581.

[6] Takai K, Nakamura K, Toki T, Tsunogai U, Miyazaki M, et al. (2008) Cell proliferation at 122°C and isotopically heavy CH_4 production by a hyperthermophilic methanogen under high-pressure cultivation. Proc Natl Acad Sci USA 105: 10949–10954.

[7] González JM, Masuchi Y, Robb FT, Ammerman JW, Maeder DL, et al. (1998) *Pyrococcus horikoshii* sp. nov., a hyperthermophilic archaeon isolated from a hydrothermal vent at the Okinawa Trough. Extremophiles 2: 123–130.

[8] González JM, Kato C, Horikoshi K (1995) *Thermococcus peptonophilus* sp. nov., a fast-growing, extremely thermophilic archaebacterium isolated from deep-sea hydrothermal vents. Arch Microbiol 164: 159–164.

[9] Grote R, Li L, Tamaoka J, Kato C, Horikoshi K, et al. (1999) *Thermococcus siculi* sp. nov., a novel hyperthermophilic archaeon isolated from a deep-sea hydrothermal vent at the Mid-Okinawa Trough. Extremophiles 3: 55–62.

[10] Meeks JC, Castenholz RW (1971) Growth and photosynthesis in an extreme thermophile, *Synechococcus lividus* (Cyanophyta). Arch Mikrobiol 78: 25–41.

[11] Ross T (1993) A philosophy for the development of kinetic models in predictive microbiology, *PhD Thesis*. Hobart: University of Tasmania.

[12] Nichols DS, Presser KA, Olley J, Ross T, McMeekin T (2002) Variation of branched-chain fatty acids marks the normal physiological range for growth in *Listeria monocytogenes*. Appl Environ Microbiol 68: 2809–2813.

[13] Benson JG (1996) Biofilms in pasteurisers: investigating growth of *Streptococcus thermophilus B.Sc. (Honours) Thesis*. Hobart: University of Tasmania.

[14] Takai K, Inoue A, Horikoshi K (1999) *Thermaerobacter marianensis* gen. nov., sp. nov., an aerobic extremely thermophilic marine bacterium from the 11000 m deep Mariana Trench. Int J Syst Evol Microbiol 49: 619–628.

[15] Hayward LJ (1990) Predictive microbiology of *Aeromonas hydrophila*: the effect of temperature and water activity on the growth of *Aeromonas hydrophila*, *B.Sc. (Honours) Thesis*. Hobart: University of Tasmania.

[16] Isaksen MF, Jorgensen BB (1996) Adaptation of psychrophilic and psychrotrophic sulfate-reducing bacteria to permanently cold marine environments. Appl Environ Microbiol 62: 408–414.

[17] Knoblauch C, Jorgensen BB (1999) Effect of temperature on sulphate reduction, growth rate and growth yield in five psychrophilic sulphate-reducing bacteria from arctic sediments. Environ Microbiol 1: 457–467.

[18] Billing E (1974) The effect of temperature on the growth of the fireblight pathogen, *Erwinia amylovora*. J Appl Bacteriol 37: 643–648.

[19] Feil H, Purcell AH (2001) Temperature-dependent growth and survival of *Xylella fastidiosa* in vitro and in potted grapevines. Plant Dis, 85: 1230–1234.

[20] Salter MA, Ross T, McMeekin TA (1998) Applicability of a model for non-pathogenic *Escherichia coli* for predicting the growth of pathogenic *Escherichia coli*. J Appl Microbiol 85: 357–364.

[21] Krist K (1997) Description and mechanisms of bacterial growth responses to water activity and compatible solutes, *PhD Thesis*. Hobart: University of Tasmania.

[22] Ingraham JL (1958) Growth of psychrophilic bacteria. J Bacteriol 76: 75–80.

[23] Barber MA (1908) The rate of multiplication of *Bacillus coli* at different temperatures. J Infect Dis 5: 379–400.

[24] Nichols DS, Greenhill AR, Shadbolt CT, Ross T, McMeekin TA (1999) Pysicochemical parameters for the growth of sea ice bacteria *Glaciecola punicea* ACAM 611^{T} and *Gelidibacter* sp. strain IC158. Appl Environ Microbiol 65: 3757–3760.

[25] Mellefont L (2000) Predictive model development and lag phase characterisation for applications in the meat industry, *PhD Thesis*. Hobart: University of Tasmania.

[26] Neumeyer K (1995) Modelling pseudomonad growth in milk and milk-based products, *MSc Thesis*. Hobart: University of Tasmania.

[27] Bowman JP, Nichols DS, McMeekin TA (1997) *Psychrobacter glacincola* sp. nov., a halotolerant, psychrophilic bacterium isolated from Antarctic sea ice. Syst Appl Microbiol 20: 209–215.

[28] Nichols DS, Olley J, Garda J, Brenner RR, McMeekin T (2000) Effect of temperature and salinity stress on growth and lipid composition of *Shewanella gelidmarina*. Appl Environ Microbiol 66: 2422–2429.

[29] Aspaly G, Stejskal V, Pekár S, Hubert J (2007) Temperature-dependent population growth of three species of stored product mites (Acari: Acaridida). Exp Appl Acarol 42: 37–46.

[30] Liu YH, Tsai JH (2000) Effects of temperature on biology and life table parameters of the Asian citrus psyllid, *Diaphorina citri* Kuwayama (Homoptera: Psyllidae). Ann Appl Biol 137: 201–206.

[31] Broufas GD, Koveos DS (2001) Development, survival and reproduction of *Euseius finlandicus* (Acari: Phytoseiidae) at different constant temperatures. Exp Appl Acarol 25: 441–460.

[32] Allen JC, Yang Y, Knapp JL (1995) Temperature effects on development and fecundity of the citrus rust mite (Acari: Eriophyidae). Environ Entomol 24: 996–1004.

[33] Roy M, Brodeur J, Cloutier C (2003) Effect of temperature on intrinsic rates of natural increase (r_m) of a coccinellid and its spider mite prey. BioControl 48: 57–72.

[34] Gotoh T, Sugimoto N, Pallini A, Knapp M, Hernandez-Suarez E, et al. (2010) Reproductive performance of seven strains of the tomato red spider mite *Tetranychus evansi* (Acari: Tetranychidae) at five temperatures. Exp Appl Acarol 52: 239–259.

[35] Sánchez-Ramos I, Castañera P (2005) Effect of temperature on reproductive parameters and longevity of *Tyrophagus putrescentiae* (Acari: Acaridae). Exp Appl Acarol 36: 93–105.

[36] Brandam C, Castro-Martínez C, Délia ML, Ramón-Portugal F, Strehaiano P (2008) Effect of temperature on *Brettanomyces bruxellensis*: metabolic and kinetic aspects. Can J Microbiol 5: 11–18.

[37] Chistyakova TA, Minkevich IG, Eroshin VK (1983) Growth of the thermotolerant yeast, *Candida valida*, on ethanol: Dependences of maximal growth rate and cell biomass yield on temperature. Eur J Appl Microbiol 18: 225–228.

[38] Salvadó Z, Arroyo-López FN, Guillamón JM, Salazar G, Querol A, et al. (2011) Temperature adaptation markedly determines evolution within the *Saccharomyces* genus. Appl Environ Microbiol 77: 2292–2302.

[39] Panagou EZ, Skandamis PN, Nychas GJE (2003) Modelling the combined effect of temperature, pH and a_w on the growth rate of *Monascus ruber*, a heat-resistant fungus isolated from green table olives. J Appl Microbiol 94: 146–156.

[40] Serra A, Strehaiano P, Taillandier P (2005) Influence of temperature and pH on *Saccharomyces bayanus* var. *uvarum* growth; impact of a wine yeast interspecific hybridization on these parameters. Int J Food Microbiol 104: 257–265.

[41] Restaino L, Bills S, Tscherneff K, Lenovich LM (1983) Growth characteristics of *Saccharomyces rouxii* isolated from chocolate syrup. Appl Environ Microbiol 45: 1614–1621.

[42] Choi WI, Ryoo MI, Kim JG (2002) Biology of *Paronychiurus kimi* (Collembola: Onychiuridae) under the influence of temperature, humidity and nutrition. Pedobiologia 46: 548–557.

[43] Rohitha BH, Penman DR (1983) Effect of temperature on the biology of bluegreen lucerne aphid, *Acyrthosiphon kondoi.* New Zeal J Zool 10: 299–308.

[44] Morgan D, Walters KFA, Aegerter JN (2001) Effect of temperature and cultivar on pea aphid, *Acyrthosiphon pisum* (Hemiptera: Aphididae) life history. Bull Entomol Res 91: 47–52.

[45] Milonas PG, Savopoulou-Soultani M (2000) Development, survivorship, and reproduction of *Adoxophyes orana* (Lepidoptera: Tortricidae) at constant temperatures. Ann Entomol Soc Am 93: 96–102.

[46] Lee JH, Ahn JJ (2000) Temperature effects on development, fecundity, and life table parameters of *Amblyseius womersleyi* (Acari: Phytoseiidae). Environ Entomol 29: 265–271.

[47] De Vis RMJ, Fuentes LE, van Lenteren JC (2002) Life history of *Amitus fuscipennis* (Hym., Platygastridae) as parasitoid of the greenhouse white fly *Trialeurodes vaporariorum* (Hom., Aleyrodidae) on tomato as function of temperature. J Appl Entomol 126: 24–33.

[48] Komazaki S (1982) Effects of constant temperatures on population growth of three aphid species, *Toxoptera citricidus* (Kirkaldy), *Aphis citricola* van der Goot and *Aphis gossypii* Glover (Homoptera: Aphididae) on citrus. Appl Entomol Zool 17: 75–81.

[49] Zamani AA, Talebi AA, Fathipour Y, Baniameri V (2006) Effect of temperature on biology and population growth parameters of *Aphis gossypii* Glover (Hom., Aphididae) on greenhouse cucumber. J Appl Entomol 130: 453–460.

[50] Aldyhim YN, Khalil AF (1993) Influence of temperature and daylength on population development of *Aphis gossypii* on *Cucurbita pepo*. Entomol Exp Appl 67: 167–172.

[51] Kocourek F, Havelka J, Beránková J, Jarošik V (1994) Effect of temperature on development rate and intrinsic rate of increase of *Aphis gossypii* reared on greenhouse cucumbers. Entomol Exp Appl 71: 59–64.

[52] Xia JY, van der Werf W, Rabbinge R (1999) Influence of temperature on bionomics of cotton aphid, *Aphis gossypii*, on cotton. Entomol Exp Appl 90: 25–35.

[53] Satar S, Kersting U, Uygun N (2005) Effect of temperature on development and fecundity of *Aphis gossypii* Glover (Homoptera: Aphididae) on cucumber. J Pest Sci 78: 133–137.

[54] Wang K, Tsai JH, Harrison NA (1997) Influence of temperature on development, survivorship, and reproduction of buckthorn aphid (Homoptera: Aphididae). Ann Entomol Soc Am 90: 62–68.

[55] Bayhan E, Ölmez-Bayhanb S, Ulusoy MR, Brown JK (2005) Effect of temperature on the biology of *Aphis punicae* (Passerini) (Homoptera: Aphididae) on pomegranate. Environ Entomol 34: 22–26.

[56] Wang JJ, Tsai JH (2000) Effect of temperature on the biology of *Aphis spiraecola* (Homoptera: Aphididae). Ann Entomol Soc Am 93: 874–883.

[57] Jandricic SE, Wraight SP, Bennett KC, Sanderson JP (2010) Developmental times and life table statistics of *Aulacorthum solani* (Hemiptera: Aphididae) at six constant temperatures, with recommendations on the application of temperature-dependent development models. Environ Entomol 39: 1631–1642.

[58] Yang P, Carey JR, Dowell RV (1994) Temperature influences on the development and demography of *Bactrocera dorsalis* (Diptera: Tephritidae) in China. Environ Entomol 23: 971–974.

[59] Wang KH, Tsai JH (1996) Temperature effect on development and reproduction of silverleaf whitefly (Homoptera: Aleyrodidae). Ann Entomol Soc Am 89: 375–384.

[60] Yang TC, Chi H (2006) Life tables and development of *Bemisia argentifolii* (Homoptera: Aleyrodidae) at different temperatures. J Econ Entomol 99: 691–698.

[61] DeLoach CJ (1974) Rate of increase of populations of cabbage, green peach, and turnip aphids at constant temperatures. Ann Entomol Soc Am 67: 332–340.

[62] Birch LC (1953) Experimental background to the study of the distribution and abundance of insects: I. The influence of temperature, moisture and food on the innate capacity for increase of three grain beetles. Ecology 34: 698–711.

[63] van Huis A, Arendse PW, Schilthuizen M, Wiegers PP, Heering H, et al. (1994) *Uscana lariophaga*, egg parasitoid of bruchid beetle storage pests of cowpea in West Africa: the effect of temperature and humidity. Entomol Exp Appl 70: 41–53.

[64] Dreyer H, Baumgärtner J (1996) Temperature influence on cohort parameters and demographic characteristics of the two cowpea coreids *Clavigralla tomentosicollis* and *C. shadabi*. Entomol Exp Appl 78: 201–213.

[65] Smith LB (1965) The intrinsic rate of natural increase of *Cryptolestes ferrugineus* (Stephens) (Coleoptera, Cucujidae). J Stored Prod Res 1: 35–49.

[66] Hosking JR (1984) The effect of temperature on the population growth potential of *Dactylopius austrinus* De Lotto (Homoptera: Dactylopiidae), on *Opuntia aurantiaca* Lindley. Aust J Entomol 23: 133–139.

[67] Sandhu HS, Nuessly GS, Webb SE, Cherry RH, Gilbert RA (2010) Life table studies of *Elasmopalpus lignosellus* (Lepidoptera:Pyralidae) on sugarcane. Environ Entomol 39: 2025–2032.

[68] Asante SK, Danthanarayana W, Heatwole H (1991) Bionomics and population growth statistics of apterous virginoparae of woolly apple aphid, *Eriosoma lanigerum*, at constant temperatures. Entomol Exp Appl 60: 261–270.

[69] Gaum WG, Giliomee JH, Pringle KL (1994) Life history and life tables of western flower thrips, *Frankliniella occidentalis* (Thysanoptera: Thripidae), on English cucumbers. Bull Entomol Res 84: 219–224.

[70] Jaramillo J, Chabi-Olaye A, Kamonjo C, Jaramillo A, Vega FE, et al. (2009) Thermal tolerance of the coffee berry borer *Hypothenemus hampei*: Predictions of climate change impact on a tropical insect pest. PLoS ONE 4: e6487.

[71] Tsoukanas VI, Papadopoulos GD, Fantinou AA, Papadoulis GT (2006) Temperature-dependent development and life table of *Iphiseius degenerans* (Acari: Phytoseiidae). Environ Entomol 35: 212–218.

[72] Wang JJ, Tsai JH, Zhao ZM, Li LS (2000) Development and reproduction of the psocid *Liposcelis bostrychophila* (Psocoptera: Liposcelididae) as a function of temperature. Ann Entomol Soc Am 93: 261–270.

[73] Wang J, Zhao Z, Li L (1998) Studies on bionomics of *Liposcelis entomophila* (Psocoptera:Liposcelididae) infesting stored product. Entomol Sinica 5: 149–158.

[74] Perdikis DC, Lykouressis DP (2002) Life table and biological characteristics of *Macrolophus pygmaeus* when feeding on *Myzus persicae* and *Trialeurodes vaporariorum*. Entomol Exp Appl 102: 261–272.

[75] Lysyk TJ (2000) Relationships between temperature and life history parameters of *Muscidifurax raptor* (Hymenoptera: Pteromalidae). Environ Entomol 29: 596–605.

[76] Lysyk TJ (2001) Relationships between temperature and life history parameters of *Muscidifurax raptorellus* (Hymenoptera: Pteromalidae). Environ Entomol 30: 982–992.

[77] Lysyk TJ (2001) Relationships between temperature and life history parameters of *Muscidifurax zaraptor* (Hymenoptera: Pteromalidae). Environ Entomol 30: 147–156.

[78] Davis JA, Radcliffe EB, Ragsdale DW (2006) Effects of high and fluctuating temperatures on *Myzus persicae* (Hemiptera: Aphididae). Environ Entomol 35: 1461–1468.

[79] Ren SX, Stansly PA, Liu TX (2002) Life history of the whitefly predator *Nephaspis oculatus* (Coleoptera: Coccinellidae) at six constant temperatures. Biol Control 23: 262–268.

[80] Kontodimas DC, Milonas PG, Stathas GJ, Economou LP, Kavallieratos NG (2007) Life table parameters of the pseudococcid predators *Nephus includens* and *Nephus bisignatus* (Coleoptera: Coccinelidae). Eur J Entomol 104: 407–415.

[81] Beckett SJ, Longstaff BC, Evans DE (1994). A comparison of the demography of four major stored grain coleopteran pest species and its implications for pest management. In: Proceedings of the 6th International Working Conference on Stored-product Protection, Volume 1, Canberra, 491–497pp.

[82] Beckett SJ, Evans DE (1994) The demography of *Oryzaephilus surinamensis* (L.) (Coleoptera: Silvanidae) on kibbled wheat. J Stored Prod Res 30: 121–137.

[83] Shi P, Li BL, Ge F (2012) Intrinsic optimum temperature of the diamondback moth and its ecological meaning. Environ Entomol 41: 714–722.

[84] Pervez A, Omkar (2004) Temperature-dependent life attributes of an aphidophagous ladybird, *Propylea dissecta*. Biocontrol Sci Techn 14: 587–594.

[85] Allsopp PG (1981) Development, longevity and fecundity of the false wireworms *Pterohelaeus darlingensis* and *P. alternatus* (Coleoptera : Tenebrionidae) I. Effect of constant temperature. Aust J Zool 29: 605–619.

[86] Rae DJ, De'ath G (1991) Influence of constant temperature on development, survival and fecundity of sugarcane mealybug, *Saccharicoccus sacchari* (Cockerell) (Hemiptera, Pseudococcidae). Aust J Zool 39: 105–122.

[87] Pakyari H, Fathipour Y, Enkegaard A (2011) Effect of temperature on life table parameters of predatory thrips *Scolothrips longicornis* (Thysanoptera: Thripidae) fed on twospotted spider mites (Acari: Tetranychidae). J Econ Entomol 104: 799–805.

[88] Turak E, Talent R, Sunnucks P, Hales DF (1998) Different responses to temperature in three closely-related sympatric cereal aphids. Entomol Exp Appl 86: 49–58.

[89] Longstaff BC, Evans DE (1983) The demography of the rice weevil, *Sitophilus oryzae* (L.) (Coleoptera: Curculionidae), submodels of age-specific survivorship and fecundity. Bull Entomol Res 73: 333–344.

[90] Murai T (2000) Effect of temperature on development and reproduction of the onion thrips, *Thrips tabaci* Lindeman (Thysanoptera: Thripidae), on pollen and honey solution. Appl Entomol Zool 35: 499–504.

[91] Wang JJ, Tsai JH (2001) Development, survival and reproduction of black citrus aphid, *Toxoptera aurantii* (Hemiptera: Aphididae), as a function of temperature. Bull Entomol Res 91: 477–487.

[92] Tsai JH, Wang K (1999) Life table study of brown citrus aphid (Homoptera: Aphididae) at different temperatures. Environ Entomol 28: 412–419.

[93] Howe RW (1962) The effects of temperature and humidity on the oviposition rate of *Tribolium castaneum* (Hbst.) (Coleoptera:Tenebrionidae). Bull Entomol Res 53: 301–310.

[94] Pratissoli D, Parra JRP (2000) Fertility life table of *Trichogramma pretiosum* (Hym., Trichogrammatidae) in eggs of *Tuta absoluta* and *Phthorimaea operculella* (Lep., Gelechiidae) at different temperatures. J Appl Entomol 124: 339–342.

[95] Chen KW, He YR, Lü YQ, Huang XLSS (2005) Effect of temperature on the population parameters of *Trichogrammatoidea bactrae* Nagaraja. Acta Ecol Sinica 25: 73–77.

[96] Naranjo SE (1993) Life history of *Trichogrammatoidea bactrae* (Hymenoptera: Trichogrammatidae), an egg parasitoid of pink bollworm (Lepidoptera: Gelechiidae), with emphasis on performance at high temperatures. Environ Entomol 22: 1051–1059.

[97] Lysyk TJ (1998) Relationships between temperature and life history parameters of *Trichomalopsis sarcophagae* (Hymenoptera: Peteromalidae). Environ Entomol 27: 488–498.

[98] Stenseng L, Skovgård H, Holter P (2003) Life table studies of the pupal parasitoid *Urolepis rufipes* (Hymenoptera: Pteromalidae) on the house fly *Musca domestica* (Diptera: Muscidae) in Denmark. Environ Entomol 32: 717–725.

[99] Sorokin C (1960) Kinetic studies of temperature effects on the cellular level. Biochim Biophys Acta 38: 197–204.
